# Dual Regulatory Role Exerted by Cyclic Dimeric GMP To Control FsnR-Mediated Bacterial Swimming

**DOI:** 10.1128/mbio.01414-22

**Published:** 2022-09-07

**Authors:** Xin Zhang, Yan Wang, Yao Wu, Zhi-Hui Yuan, Zhen Cai, Wei Qian, Xin Ge, Fang-Fang Wang

**Affiliations:** a State Key Laboratory of Plant Genomics, Institute of Microbiology, Chinese Academy of Sciences, Beijing, China; b CAS Center for Excellence in Biotic Interactions, University of Chinese Academy of Sciences, Beijing, China; c School of Life Science, Hebei University, Baoding, China; d School of Life Sciences, Yunnan University, Kunming, China; e Aviation General Hospital of China Medical University, Beijing, China; Biozentrum/University of Basel; The Ohio State University

**Keywords:** c-di-GMP, ferrous iron, flagella, phosphodiesterase, swimming

## Abstract

Bacterial motility has great medical and ecological significance because of its essential role in bacterial survival and pathogenesis. Cyclic dimeric GMP (c-di-GMP), a second messenger in bacteria, is the predominant regulator of flagellar synthesis and motility and possesses turnover mechanisms that have been thoroughly investigated. Therefore, much attention has been focused on identifying the upstream stimulatory signals and downstream modules that respond to altered c-di-GMP levels. Here, we systematically analyzed c-di-GMP cyclases and phosphodiesterases in Stenotrophomonas maltophilia to screen for motility regulators. Of these enzymes, we identified and characterized a new phosphodiesterase named SisP, which was found to facilitate bacterial swimming upon stimulation with ferrous iron. SisP-mediated degradation of c-di-GMP leads to FsnR-dependent transcription of flagellar genes. Remarkably, c-di-GMP controls FsnR via two independent mechanisms: by direct binding and indirectly by modulating its phosphorylation state. In this study, we deciphered a novel “one stone, two birds” regulatory strategy of c-di-GMP and uncovered the signal that stimulates c-di-GMP hydrolysis. Facilitation of bacterial swimming motility by ferrous iron might contribute to the higher risk of bacterial infection in acutely ill patients.

## INTRODUCTION

Over the past several decades, the opportunistic pathogen Stenotrophomonas maltophilia, a Gram-negative bacterium, has become a great threat to human health, typically infecting immunosuppressed or immunocompromised patients, especially those in intensive care units. Infection usually results in urinary tract infection, meningitis, bacteremia or hemorrhagic pneumonia. The overall mortality of infected patients ranges from 35 to 75%, while the mortality of hemorrhagic pneumonia caused by S. maltophilia is nearly 100%, mainly due to its intrinsic resistance to multiple antibiotics, including cephalosporins and meropenem (1, 2). The effectiveness of trimethoprim-sulfamethoxazole, the antibiotic treatment of choice, is diminishing (3, 4). This urgent situation calls for novel strategies to control S. maltophilia infection.

Swimming motility is a critical aspect of bacterial pathogenesis and is required for nutrition acquisition, movement toward attractants, host invasion, and colonization. It has long been recognized that the control of bacterial motility is a feasible approach to assist the treatment of bacterial infection, while avoiding the occurrence of antibiotic resistance (5–8). The flagellum is the most important organelle for bacterial swimming. It is composed of a reversible rotary molecular motor anchored on the cell envelope, an extracellular filament that acts as a propeller for swimming motility, and a hook that connects the motor and the filament (9, 10). A bacterium synthesizes and assembles the flagella before the motility of the flagella can be regulated (11). The synthesis and assembly of a flagellum responds to environmental changes and occurs in a stepwise fashion through an organized transcriptional hierarchy of flagellar gene expression. This hierarchy contains three to four classes of flagellar genes, on top of which a sole master regulator initiates and controls expression of flagellar genes. This master regulator, including FlrA (also named FleQ) in Pseudomonas aeruginosa and Vibrio cholerae, and FlaK in Vibrio parahaemolyticus, varies in different species, and its expression and activity are tightly regulated by regulatory proteins or molecules whose cellular level or activity is usually modulated in response to changes in the environment (12–15). Cyclic dimeric GMP (bis-[3′, 5′]-cyclic dimeric GMP [c-di-GMP]), a second messenger, is one of such molecules whose cellular level is fine-tuned in response to environmental stimuli and plays an important role in regulating flagellar synthesis. A higher cellular level of c-di-GMP is usually associated with few or even no flagella or reduced flagellar motility (16, 17). Efforts to uncover the underlying mechanisms of c-di-GMP in the regulation of flagellar synthesis have yielded results. Reportedly, c-di-GMP directly binds and inhibits the activities of the master regulators FlaK and FlrA, thereby decreasing the expression of flagellar genes, as well as flagellum numbers (11, 16). However, considering the variety of master regulators found in different bacterial species, c-di-GMP may have multiple regulatory modes. Otherwise, c-di-GMP indirectly modulates the expressions or activities of the mater regulator like FlrA via binding and regulating the activities of their upstream regulatory proteins, although the mechanisms employed by many such regulatory proteins to control expressions or activities of the master regulators are poorly understood. For instance, the receptor histidine kinase (HK) RavS unbound by c-di-GMP has a higher phosphorylation level and promotes flagellar synthesis in Xanthomonas campestris, for which the details of RavS regulating the master regulator FlrA are unknown (17). SadB, a modified HD-GYP domain containing protein, binds c-di-GMP to regulate expression of AlgU, an upstream sigma factor that controls FlrA expression in Pseudomonas fluorescens, but further regulatory details of how c-di-GMP modulates the activity of SadB remain unknown (18). In S. maltophilia, there is a limited understanding of how c-di-GMP regulates flagellar synthesis and flagella numbers. The only reported regulatory protein in S. maltophilia is BsmR, an EAL domain-containing phosphodiesterase (PDE) that positively regulates expression of *fsnR* to facilitate flagellar synthesis (19). The *fsnR* gene, for which the genomic deletion abolishes flagellar synthesis, encodes a transcription regulator that directly binds to the promoters of *fliD* and *cheV* to promote their expression. As reported in other species, FliD caps the distal end of the flagellar filament to protect it by preventing the release of flagellin (20, 21), while CheV is a chemotactic connexin that can replace or enhance the function of CheW, an adaptor for accommodating specific chemoreceptors within the chemotaxis signaling complex, as reported previously (22). However, their roles in S. maltophilia have not been reported. In addition, FsnR positively regulates the expression of *fliC* and *fliE*, the flagellar component genes, and *flrA*, the yet-to-be-studied putative master regulator gene of flagellar gene expression (23), suggesting that FsnR is upstream of FlrA in regulating flagellar synthesis and flagellum numbers. It is interesting to find that deletion of *bsmR* significantly decreases the expression of *fsnR* but barely affects bacterial swimming motility, suggesting that the activity of FsnR rather than its expression level plays the more important role in regulating flagellar synthesis. In addition, two putative c-di-GMP turnover enzymes, RavR and RpfG, have been studied in X. campestris, and the regulatory mechanisms of their activities and the following signaling regulations have been elucidated. Both proteins are response regulators (RRs), which pair with cognate receptor HKs to constitute the significant two-component signal transduction systems in bacteria. Typically, an HK detects a specific signal, autophosphorylates on its conserved His residue, and transfers the phosphoryl group to its cognate RR. The RR accepts the phosphoryl group through the conserved Asp residue within the REC domain, and performs downstream regulation through its output domain (24, 25). The phosphodiesterase activity of RavR, a GGDEF-EAL domain-containing protein, is activated by phosphorylation performed by its cognate HKs RavA and RavS and is putatively stimulated by a low-oxygen tension signal (26, 27), while that of RpfG, an HD-GYP domain-containing protein, is stimulated through phosphorylation by its cognate HK RpfC in response to the quorum-sensing (QS) signal, diffusible signal factor (DSF) (28, 29). Both RavR and RpfG degrade c-di-GMP to release its inhibition of the activity of the global transcription factor Clp, which binds the promoter and activates the expression of *fleQ* in X. campestris (30, 31). Cheng et al. reported that RavS with a higher phosphorylation level plays a positive role in regulating flagellar synthesis through an unknown mechanism. Moreover, during this regulation, RavR, by accepting the phosphoryl group from RavS, decreases the phosphorylation level of RavS and bacterial swimming motility, which explains the promotion in swimming motility by genomic *ravR* deletion (17). However, further investigations of both RavR and RpfG in S. maltophilia are still needed to examine whether their enzymatic activities and regulatory details are conserved in S. maltophilia.

c-di-GMP has been investigated for decades and great progress has been made in understanding its turnover by diguanylate cyclases (DGCs) and PDEs. DGCs contain the conserved GGDEF (Gly-Gly-Asp-Glu-Phe) domain and generates c-di-GMP from two molecules of GTP, while PDEs contain the EAL (Glu-Ala-Leu) or HD-GYP domain and hydrolyze c-di-GMP into pGpG or two molecules of GMP, respectively (32–34). Research emphasis has largely shifted to the identification of upstream signals that regulate activities of the DGCs and PDEs, and the characterization of downstream modules, including identification of c-di-GMP effectors and regulatory mechanisms of the signaling that responds to altered c-di-GMP levels. Here, we systematically analyzed all of the putative enzymes involved in c-di-GMP turnover in S. maltophilia. We identified and further investigated a newly identified phosphodiesterase named SisP (S. maltophilia iron-sensing PDE), and uncovered its stimulatory signal and downstream module. Interestingly, in this module, c-di-GMP was found to exert two-layered regulation on the activity of FsnR by modulating its phosphorylation level while simultaneously binding FsnR directly. This finding reflects a direct and indirect model of c-di-GMP in regulation of the activity of its effector and implies how little is known about c-di-GMP regulation. Moreover, the signal detected by SisP was identified as ferrous iron, which is commonly found at the sites of cuts and other wounds. We propose that the process by which SisP detects ferrous iron to facilitate bacterial swimming motility might also be utilized to recognize cuts or wounds, allowing bacteria to enter the human body.

## RESULTS

### Systematic mutational analysis of genes encoding GGDEF, EAL, and HD-GYP domain-containing proteins in the opportunistic pathogen *S. maltophilia*.

Simple Modular Architecture Research Tool (SMART) (35) analyses were used to find putative c-di-GMP turnover enzymes. The results showed that the genome of S. maltophilia encodes 33 such proteins: 17 GGDEF domain-containing proteins, 6 EAL domain-containing proteins, 2 HD-GYP domain-containing proteins, and 8 GGDEF and EAL domain-containing proteins (see Fig. S1 in the supplemental material). The large number of putative c-di-GMP turnover enzymes suggested that c-di-GMP plays a significant role in S. maltophilia and its levels are fine-tuned. With the exception of BsmR, none of these proteins have been studied in this species.

10.1128/mbio.01414-22.2 FIG S1 Domain organization of DGCs and PDEs in S. maltophilia. Protein structures were predicted by SMART analyses. Black vertical bars represent transmembrane helices. Names of protein domains in accordance with those in the SMART database. Numbered open reading frames (ORFs) present proteins encoded by the corresponding genomic gene. Proteins that are involved in bacterial swimming motility and lack any recognizable sensor domain are highlighted by the blue color. Download FIG S1, TIF file, 1.3 MB. Copyright © 2022 Zhang et al. 2022 Zhang et al. https://creativecommons.org/licenses/by/4.0/ This content is distributed under the terms of the Creative Commons Attribution 4.0 International license.

**FIG 1:**
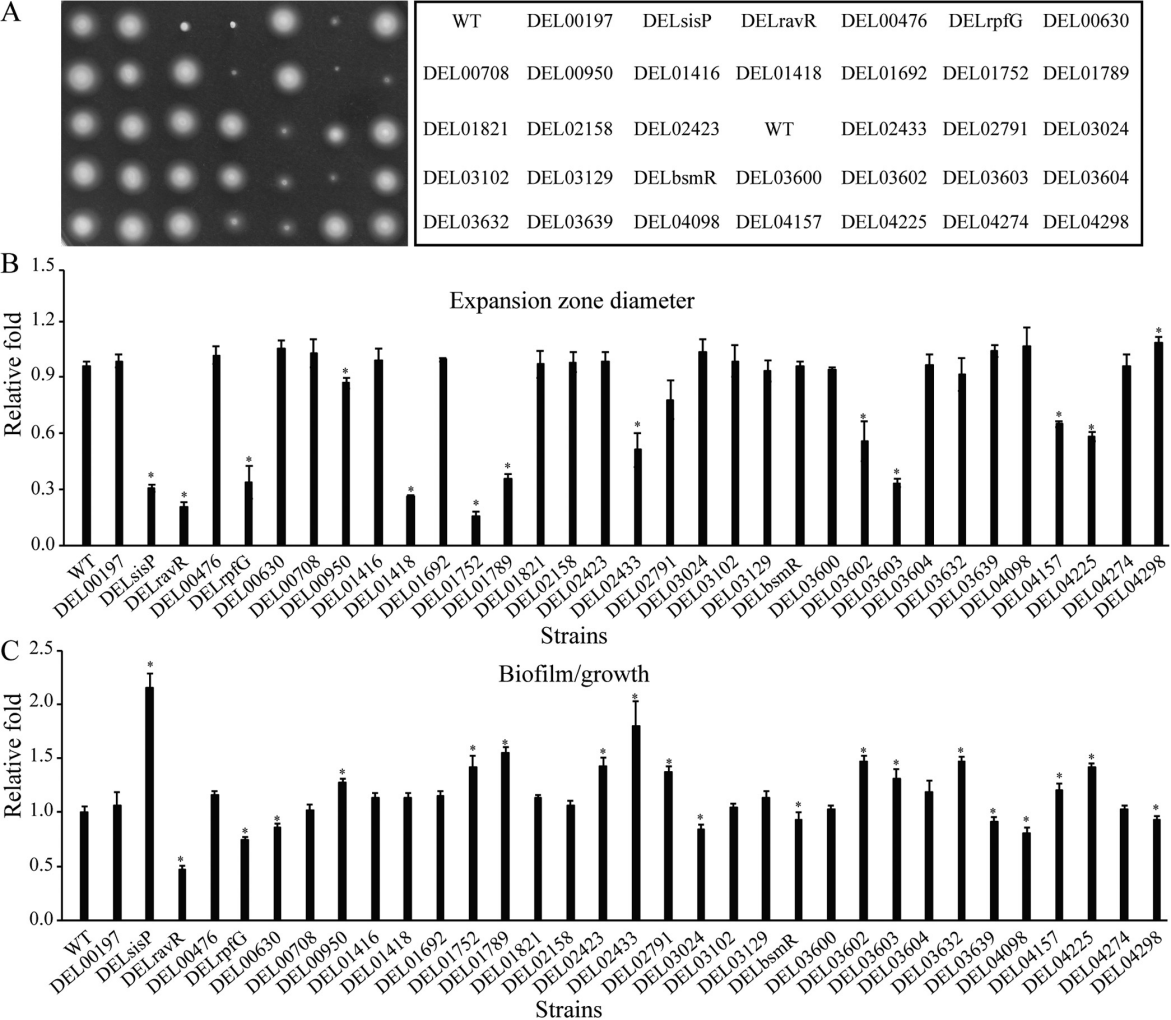
Systematic analyses of swimming motility and biofilm formation ability of all S. maltophilia *DGC* and *PDE* mutant strains. (A) Swimming motility (left panel) of each strain (right panel). “DEL” followed by a number represents the mutant with insertional inactivation of the corresponding gene. (B) Quantification of the expansion zone diameters corresponding to panel A and calculation of fold changes relative to diameters in the WT strain. (C) Quantification of biofilm relative that produced by the WT strain. Data represent three biological replicates. Error bars represent standard deviations (*n* ≥ 3 biological replicates). Student’s *t* tests were performed (*, *P* ≤ 0.05).

To systematically screen for enzymes that control bacterial swimming motility, we constructed mutants of all 33 genes through insertional inactivation that might have putative polar effects and investigated their swimming motility. As shown in Fig. 1A and B, 12 mutant strains showed deficiency in bacterial swimming motility, and one showed promotion, suggesting that these 13 genes might function in regulation of bacterial swimming motility. However, mutation of a DGC or a PDE will not necessarily change the cellular c-di-GMP level and the bacterial swimming motility for degeneration or inactivation or redundancy of these enzymes; thus, further analyses are still needed to verify the individual roles of each gene in bacterial swimming motility. Among the proteins encoded by genes that correspond to these 13 mutants, RpfG positively regulates bacterial swimming motility for the significant decrease in bacterial expansion zone caused by its gene insertional inactivation (Fig. 1A and B), which is consistent with the regulatory role of its homolog in X. campestris. However, it was interesting to find that RavR plays a putative positive role in regulating bacterial swimming motility for the absolute collapse in swimming motility due to insertional inactivation of the *ravR* gene (Fig. 1A and B). This result suggests the putative specificity of S. maltophilia in regulating bacterial swimming motility in comparison with X. campestris for the oppositely negative role of RavR in regulating the swimming motility of X. campestris. The orf00950, orf01418, orf01752, orf03602, orf03603, orf04098, orf04225, and orf04298 proteins lack any recognizable sensor domains (see Fig. S1, highlighted by the blue color), suggestive of the enzymatic stimulation by covalent modification or interaction with other regulatory proteins or reception of small molecules by currently not defined domains. The orf04225, for example, is an RR, suggesting that its enzymatic activity may be modulated by phosphorylation. Three others possess recognizable sensor domains (see Fig. S1, PAC-PAS-PAC-PAC of orf00431 (SisP), FN3 (the tenth fibronectin type III repeat containing an RGD cell recognition sequence) of orf01789, and PAS of orf02433), suggestive of direct induction by specific signals. Of these three proteins, SisP has the most complex protein structure PAC-PAS-PAC-PAC-GGDEF-EAL, indicating that it may exhibit flexibility and complexity in signal perception and regulation. Therefore, we chose to study SisP further.

We performed biofilm formation analysis that identified 20 mutants as being involved in the control of biofilm formation; half of the mutants showed increased amounts of biofilm, and half showed decreased amounts (Fig. 1C). Among the 13 mutant strains with altered bacterial swimming motility, two strains with deficiency in swimming motility also showed deficiency in biofilm formation ability (DELravR and DELrpfG), while one (DEL01418) with a deficiency in swimming motility behaved similarly to the wild-type strain (WT) in biofilm formation ability. Therefore, bacteria might employ a separate set of genes to regulate their swimming motility, with or without reverse regulation of biofilm formation.

### SisP degrades c-di-GMP to facilitate bacterial swimming motility.

**FIG 2:**
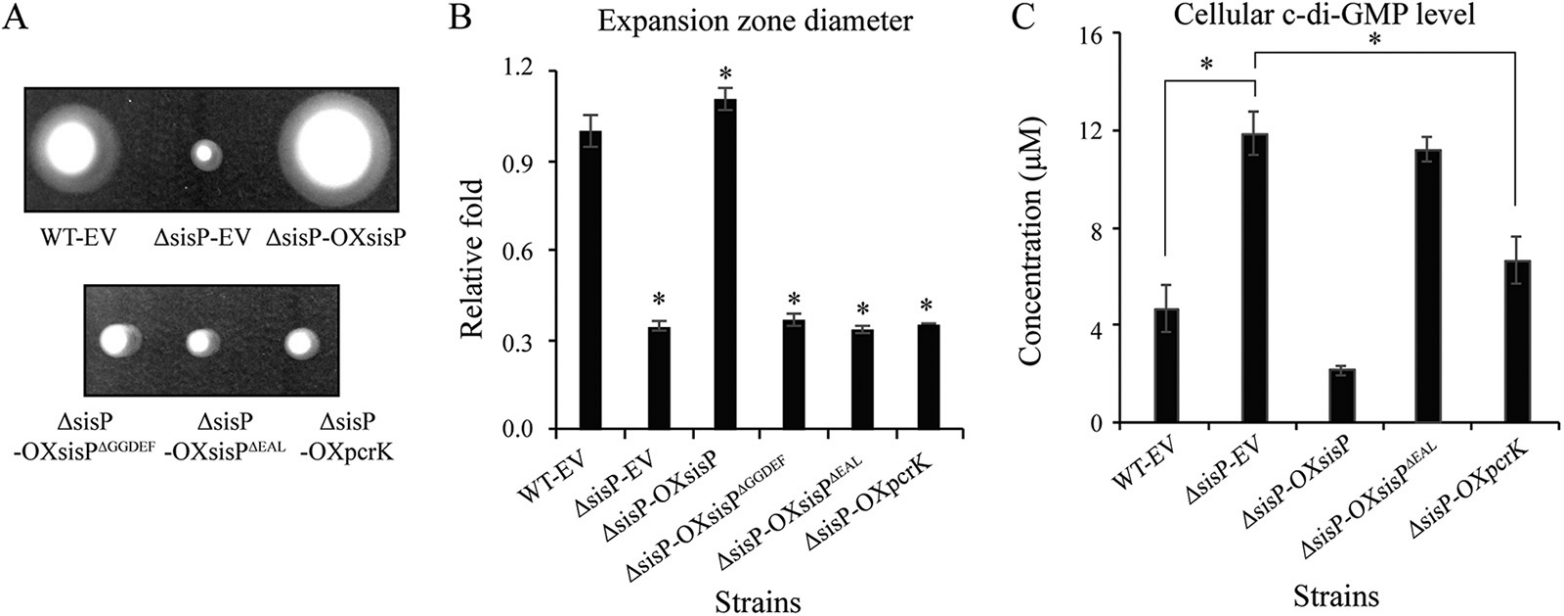
SisP degrades c-di-GMP to facilitate bacterial swimming motility in S. maltophilia. (A) Representative swimming motility of the indicated strains, demonstrating positive regulation by SisP and the necessity of both GGDEF and EAL domains. (B) Quantification of the expansion zone diameters corresponding to panel A and calculation of fold changes relative to diameters in the WT strain. (C) LC-MS/MS measurement of cellular c-di-GMP levels demonstrating significant SisP-mediated decreases in the cellular c-di-GMP level. WT-EV, WT strain bearing the EV pBBR1MCS2; ΔsisP-EV, genomic *sisP* deletion bearing pBBR1MCS2; ΔsisP-OXsisP, constitutive expression of *sisP* by the vector pBBR1MCS2::*sisP* in the background of genomic *sisP* deletion; ΔsisP-OXsisP^ΔGGDEF^, constitutive expression of *sisP* lacking sequences encoding the GGDEF domain (*sisP^ΔGGDEF^*) in the background of genomic *sisP* deletion; ΔsisP-OXsisP^ΔEAL^, constitutive expression of *sisP* lacking sequences encoding the EAL domain (*sisP^ΔEAL^*) in the background of genomic *sisP* deletion; ΔsisP-OXpcrK, constitutive expression of *pcrK* in the background of genomic *sisP* deletion. Data represent three biological replicates. Error bars represent standard deviations. Student’s *t* tests were performed (*, *P* ≤ 0.05).

We continued our investigation of SisP by verifying its regulatory role using genetic analysis to exclude the possibility of polar effects in the insertional inactivation mutant of *sisP*. We constructed the following strains and assessed their swimming motility: an in-frame deletion mutation of *sisP* (ΔsisP-EV, a Δ*sisP* strain bearing the empty vector [EV] pBBR1-MCS2, as a negative control for the complementation) and complementary strains with constitutive expression of *sisP or sisP* lacking sequence encoding the GGDEF domain (*sisP^ΔGGDEF^*, deletion of the entire GGDEF domain encoding sequences [amino acids 466 to 638]) or the EAL domain (*sisP^ΔEAL^*, deletion of the entire EAL domain encoding sequences [amino acids 648 to 894]; ΔsisP-OXsisP, ΔsisP-OXsisP^ΔGGDEF^, and ΔsisP-OXsisP^ΔEAL^). The expansion zone of ΔsisP-EV was significantly decreased to 0.31-fold that of the WT-EV, while the expansion zone of the complementary strain ΔsisP-OXsisP surpassed even that of the WT strain (Fig. 2A and B). These results suggested that *sisP* positively regulates bacterial swimming motility. To confirm the enzymatic activity employed by SisP in regulating bacterial swimming motility, we measured the cellular c-di-GMP levels of the WT-EV, ΔsisP-EV, and ΔsisP-OXsisP strains. As expected, there was a significant increase in cellular c-di-GMP level of the ΔsisP-EV strain, 2.6-fold that of the WT-EV strain (Fig. 2C). In the ΔsisP-OXsisP strain, a significant decrease in the c-di-GMP level was identified, 0.4-fold that of the WT-EV strain, possibly because of overproduction of SisP by the pBBR1MCS2::*sisP* vector (Fig. 2C). These results suggested that the phosphodiesterase activity is employed by SisP to regulate bacterial swimming motility. This notion was further supported by the observation that deletion of the EAL domain of SisP in the ΔsisP-OXsisP^ΔEAL^ strain, which constitutively expresses the recombinant SisP^ΔEAL^ protein yielded phenotypes of swimming motility and cellular c-di-GMP level that were similar to those of the ΔsisP-EV strain (Fig. 2A to C). To examine the role of the GGDEF domain of SisP in regulating bacterial swimming motility, we investigated swimming motility of the ΔsisP-OXsisP^ΔGGDEF^ strain and found that deletion of the GGDEF domain of SisP also significantly decreased bacterial swimming motility (Fig. 2A and B), which was attributed to instability of cellular SisP^ΔGGDEF^ because of the lack of detectable band for the SisP^ΔGGDEF^ protein in the Western blot of the ΔsisP-OXsisP^ΔGGDEF^ strain (see Fig. S2). Moreover, as shown in Fig. S2, more SisP^ΔEAL^ protein was observed in the ΔsisP-OXsisP^ΔEAL^ strain than SisP protein in the ΔsisP-OXsisP strain. In addition, expressing the PDE PcrK from X. campestris in the *sisP* deletion mutant (the ΔsisP-OXpcrK strain) reduced c-di-GMP levels but failed to rescue cell motility (Fig. 2A to C), arguing that motility control by SisP is highly specific, possibly by interaction with or being spatially close to its downstream targets.

10.1128/mbio.01414-22.3 FIG S2 The GGDEF domain plays a positive role in regulating stability of SisP, while the EAL and sensor domains play negative roles. Western blotting showing the intracellular stability of His_6_-tagged SisP and recombinant proteins SisP^ΔGGDEF^, SisP^ΔEAL^, and SisP^Δsensor^. Total bacterial proteins extracted from strains cultured overnight were used for the analysis. RNA polymerase α-subunit was used as a loading control. MW, the expected molecular mass of SisP and its truncated proteins in the corresponding strains. Data represent at least three biological repeats. Download FIG S2, TIF file, 0.4 MB. Copyright © 2022 Zhang et al. 2022 Zhang et al. https://creativecommons.org/licenses/by/4.0/ This content is distributed under the terms of the Creative Commons Attribution 4.0 International license.

Consistently, the ΔsisP-EV and ΔsisP-OXsisP^ΔEAL^ strains showed stagnant or Brownian motion-like behaviors, while the WT-EV and ΔsisP-OXsisP strains exhibited relatively normal swimming behaviors under light microscopy (see Video S1). We further explored the regulatory role of SisP through phenotype screening and found that both biofilm formation ability and the MIC of meropenem (a broad-spectrum antibiotic of the carbapenem class) were significantly increased in ΔsisP-EV compared to WT-EV and ΔsisP-OXsisP (see Fig. S3A and B), suggesting that SisP negatively regulates bacterial biofilm formation and increases meropenem resistance. The possibility that deletion of sisP decreases swimming motility and enhances biofilm formation and meropenem resistance via modulating the growth rate was excluded since deletion of *sisP* did not significantly change bacterial growth rates, albeit at high concentrations, SisP accelerated growth rates at the early stage (see Fig. S4).

10.1128/mbio.01414-22.4 FIG S3 SisP negatively regulates bacterial biofilm formation ability and MIC of meropenem. (A) Crystal violet-stained biofilms formed by the indicated bacterial strains (upper panel) and quantification of fold changes relative to amounts of biofilm in the WT-EV strain using the OD_590_/OD_600_ ratio (lower panel). (B) MICs of meropenem of the bacterial strains, indicating that SisP negatively regulates bacterial survival under meropenem. Cons, concentrations. Data represent at least three biological repeats. Error bars represent standard deviations. Student’s *t* tests were performed (*, *P* ≤ 0.05; n.s., no significant differences). Download FIG S3, TIF file, 1.1 MB. Copyright © 2022 Zhang et al. 2022 Zhang et al. https://creativecommons.org/licenses/by/4.0/ This content is distributed under the terms of the Creative Commons Attribution 4.0 International license.

10.1128/mbio.01414-22.5 FIG S4 Deletion of genomic *sisP* does not affect bacterial growth rate. Growth curves for bacterial strains cultured in rich LB medium are shown. Data represent three biological repeats. Error bars represent standard deviations. Download FIG S4, TIF file, 0.2 MB. Copyright © 2022 Zhang et al. 2022 Zhang et al. https://creativecommons.org/licenses/by/4.0/ This content is distributed under the terms of the Creative Commons Attribution 4.0 International license.

Taken together, our results suggested that SisP degrades c-di-GMP through its EAL domain to facilitate swimming motility and negatively regulates bacterial biofilm formation and increases meropenem resistance. In addition, the GGDEF domain appears to be critical for protein stability of intracellular SisP proteins in S. maltophilia cells, while the EAL domain exerts negative effects on stability of intracellular SisP protein.

### Detection of ferrous iron specifically activates SisP phosphodiesterase activity toward c-di-GMP.

**FIG 3:**
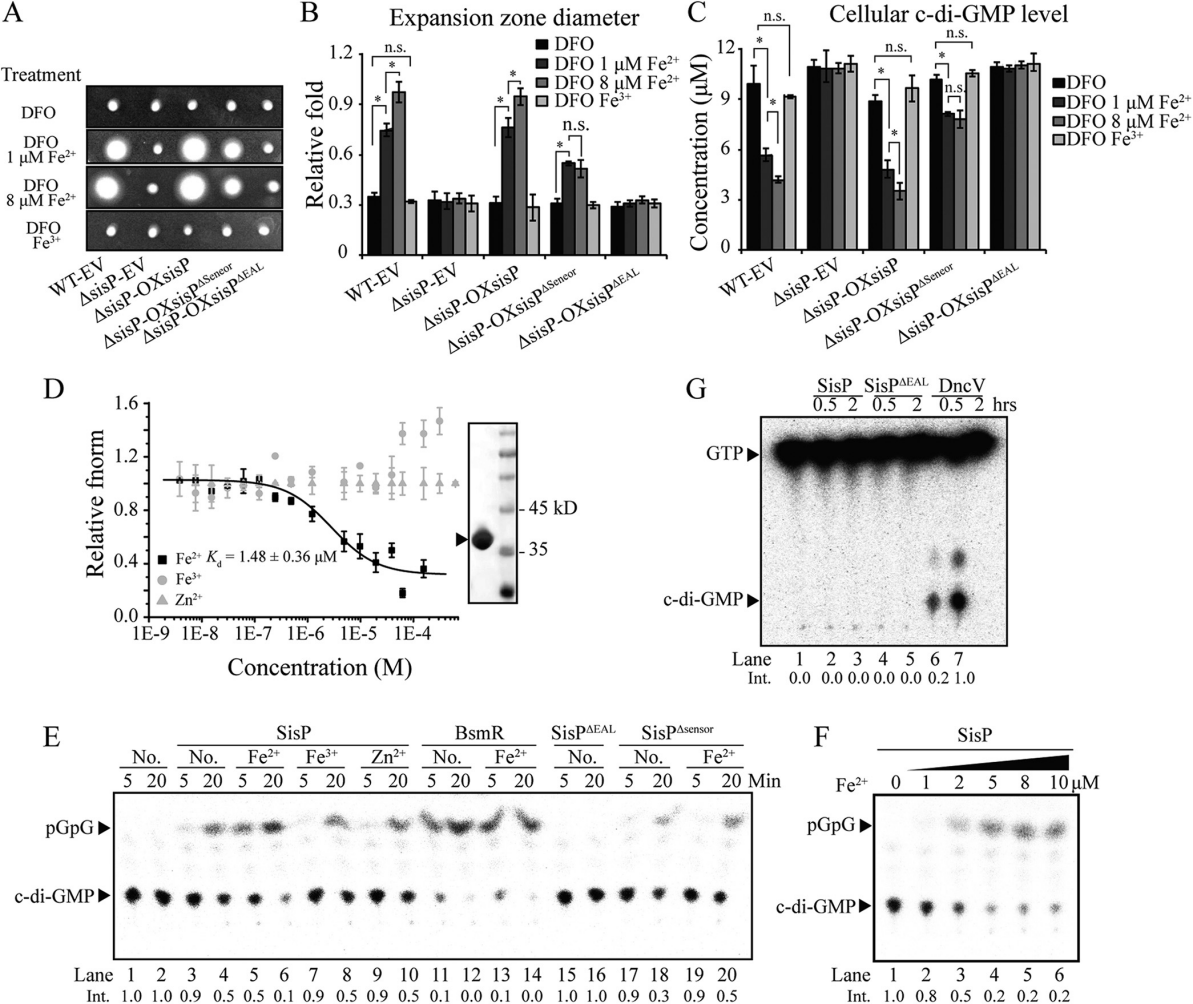
Ferrous iron stimulates SisP to activate its phosphodiesterase activity and thereby facilitate bacterial swimming motility in S. maltophilia. (A) SisP is specifically and positively regulated by ferrous iron to facilitate bacterial swimming motility. Swimming motility of the indicated strains grown in LB medium containing 0.15% agar under the following conditions: (i) pretreatment with DFO for 2 h to deplete iron (DFO) or (ii) pretreatment with DFO for 2 h, followed by the addition of 1 μM FeSO_4_ (DFO 1 μM Fe^2+^), 8 μM FeSO_4_ (DFO 8 μM Fe^2+^), or 8 μM FeCl_3_ (DFO Fe^3+^). The ΔsisP-OXsisP^Δsensor^ strain constitutively expresses SisP with the sensor domains deleted (pBBR1MCS2::*sisP^Δsensor^*) in a background of genomic *sisP* deletion. (B) Measurement and quantification of the expansion zone diameters corresponding to panel A and calculation of the fold changes relative to diameters in the WT strain. (C) SisP specially responds to ferrous iron to decrease the cellular c-di-GMP level. LC-MS/MS measurements of cellular c-di-GMP levels for each strain, cultured with or without Fe^2+^ or Fe^3+^, in LB medium pretreated with DFO for 2 h. (D) MST measurement of direct interaction between the sensor domains of SisP and three ions (ferrous, ferric, and zinc), showing a specificity for ferrous iron. Fluorescein-labeled SisP protein (20 nM) was incubated with increasing concentrations of the indicated ion. The dissociation constant (*K_d_*) was quantified to estimate the binding affinity of direct interactions. The purity of the sensor domains of SisP was examined, as shown in the right panel. (E) Ferrous iron specifically stimulates the PDE activity of SisP through its sensor domains. Analysis of PDE activities of the indicated proteins incubated with c-di-GMP in the presence of ferrous iron, zinc ion, or ferric ion, demonstrating specific stimulation of SisP by ferrous iron through the sensor domains. Reactions were stopped at the indicated times and subjected to TLC to separate c-di-GMP and the product pGpG. Purified BsmR, an EAL domain-containing protein, was used as a control. (F) TLC analysis showing dose-dependent activation of SisP incubated with c-di-GMP for 20 min in the presence of ferrous iron at stepwise increases in concentration. (G) TLC analysis showing no detectable DGC activity of SisP following incubation with GTP for 0.5 and 2 h, respectively. The DGC DncV from E. coli was used as the positive control. The “Int.” in panels E, F, and G represents the intensity of the c-di-GMP band measured by ImageJ software. The lane number and the “Int.” are below the panel. Data represent at least three biological repeats. Error bars represent standard deviations. Student’s *t* tests were performed (*, *P* ≤ 0.05; n.s., no significant differences).

Next, we constructed the ΔsisP-OXsisP^Δsensor^ strain, which constitutively expresses recombinant SisP protein lacking the PAC-PAS-PAC-PAC domains (SisP^Δsensor^, deletion of most sequences of the sensor domains [amino acids 226 to 375] to avoid the potential effect of the deletion on the enzymatic activity of the recombinant SisP), and performed Western blot analysis to examine its intracellular stability. The larger amount of SisP^Δsensor^ compared to SisP suggested that SisP^Δsensor^ is more stable in S. maltophilia cells (see Fig. S2). To identify the specific signal detected by the SisP PAC-PAS-PAC-PAC domains, we conducted signal screening assays. We found that depletion of both ferric and ferrous irons using the specific chelator deferoxamine mesylate salt (DFO) (36, 37) disabled swimming in all five strains—WT-EV, ΔsisP-EV, ΔsisP-OXsisP, ΔsisP-OXsisP^Δsensor^, and ΔsisP-OXsisP^ΔEAL^—suggestive of the role of ferrous or ferric iron or both in activating SisP to facilitate bacterial swimming. Supplementation with increasing concentrations of ferrous iron led to a gradual improvement in bacterial swimming motility in the WT-EV and ΔsisP-OXsisP strains but not in the ΔsisP-EV and ΔsisP-OXsisP^ΔEAL^ strains, which was consistent with the gradual decrease in cellular c-di-GMP levels in the WT EV and ΔsisP-OXsisP strains, along with the increase in the concentrations of ferrous iron (Fig. 3A to C). In contrast, there was no improvement in swimming motility of any of the strains when ferric ion was added into the DFO-pretreated medium. These results suggested that SisP directly perceives ferrous iron to degrade c-di-GMP, thus facilitating bacterial swimming motility. Moreover, compared to the similarity of the WT-EV and ΔsisP-OXsisP^Δsensor^ strains in the swimming motility, the expansion zone diameter of the ΔsisP-OXsisP^Δsensor^ strain growing in medium supplemented with 1 μM ferrous iron significantly decreased to 52.4% that of the WT-EV strain, and no improvement was identified when 8 μM ferrous iron was supplemented into the DFO-pretreated medium (Fig. 3A and B). This phenotype was consistent with that of the cellular c-di-GMP level in the ΔsisP-OXsisP^Δsensor^ strain, which showed no significant difference in c-di-GMP levels in the ΔsisP-OXsisP^Δsensor^ strain growing in medium supplemented with a lower (1 μM) or higher (8 μM) level of ferrous iron (Fig. 3C). These results demonstrated that the PAC-PAS-PAC-PAC sensor domains of SisP are required for the perception of ferrous iron. In addition, the strong stability of the SisP^Δsensor^ protein might have contributed to the detectable recovery in swimming motility and decrease in the cellular c-di-GMP level of the ΔsisP-OXsisP^Δsensor^ strain growing in medium supplemented with ferrous iron (Fig. 3A to C).

To further test for a direct interaction between the sensor domains (amino acids 151 to 486 of SisP) and ferrous iron, we performed microscale thermophoresis (MST) assays. As expected, the sensor domains directly bound to ferrous iron with a moderate binding affinity (*K_d_* = 1.48 ± 0.36 μM, which is larger than 0.5 μM) (38), whereas no direct interaction was detected between the sensor domains and either of the negative controls (zinc ion and ferric iron) (Fig. 3D), demonstrating the sensors domains are involved in the ferrous iron perception by SisP. To investigate the putative regulatory role of ferrous iron on the enzymatic activity of SisP, we expressed and purified the SisP protein and performed enzymatic activity analyses *in vitro*. A remarkable decrease in c-di-GMP level was observed after incubation of SisP with c-di-GMP for 20 min, which coincided with the appearance of the hydrolytic product pGpG (Fig. 3E, lanes 3 and 4 compared to lanes 1 and 2). In contrast, there was no recognizable decrease in c-di-GMP or appearance of pGpG when a negative control, the recombinant SisP^ΔEAL^ protein, was used instead of WT SisP protein (Fig. 3E, lanes 15 and 16 compared to lanes 1 and 2). Moreover, a further decrease and increase in the c-di-GMP and pGpG levels, respectively, were observed following the addition of ferrous iron, but not the negative control zinc ion or ferric iron, to the reaction system with SisP (Fig. 3E, lanes 5 and 6, 7 and 8, and 9 and 10 compared to lanes 3 and 4). The lack of any detectable enhancement of PDE activity of the BsmR protein (Fig. 3E, lanes 13 and 14 compared to lanes 11 and 12), demonstrated the specificity of ferrous iron in regulating the PDE activity of SisP. Furthermore, when SisP^Δsensor^ was used in the reaction system, there was no further decrease in c-di-GMP following the addition of ferrous iron (Fig. 3E, lanes 19 and 20 compared to lanes 17 and 18), demonstrating that the sensor domains of SisP are involved in ferrous iron perception. In addition, the PDE activity of the SisP protein was activated by ferrous iron in a dose-dependent manner, because a significant decrease in the c-di-GMP level, together with a significant increase in the pGpG level, was observed along with increasing concentrations of ferrous iron (Fig. 3F). Taken together, ferrous iron specifically binds and stimulates SisP to degrade c-di-GMP in a dose-dependent manner, for which the sensor domains of SisP are required. However, based on the lack of c-di-GMP products, there was no detectable DGC activity in SisP or SisP^ΔEAL^ protein using the *in vitro* assay (Fig. 3G). In contrast, there was a remarkable increase in the production of c-di-GMP over time when the DncV protein from Escherichia coli was used as the positive control in the reaction system (Fig. 3G).

### SisP positively regulates flagellar gene expression and facilitates flagellar synthesis and assembly.

**FIG 4:**
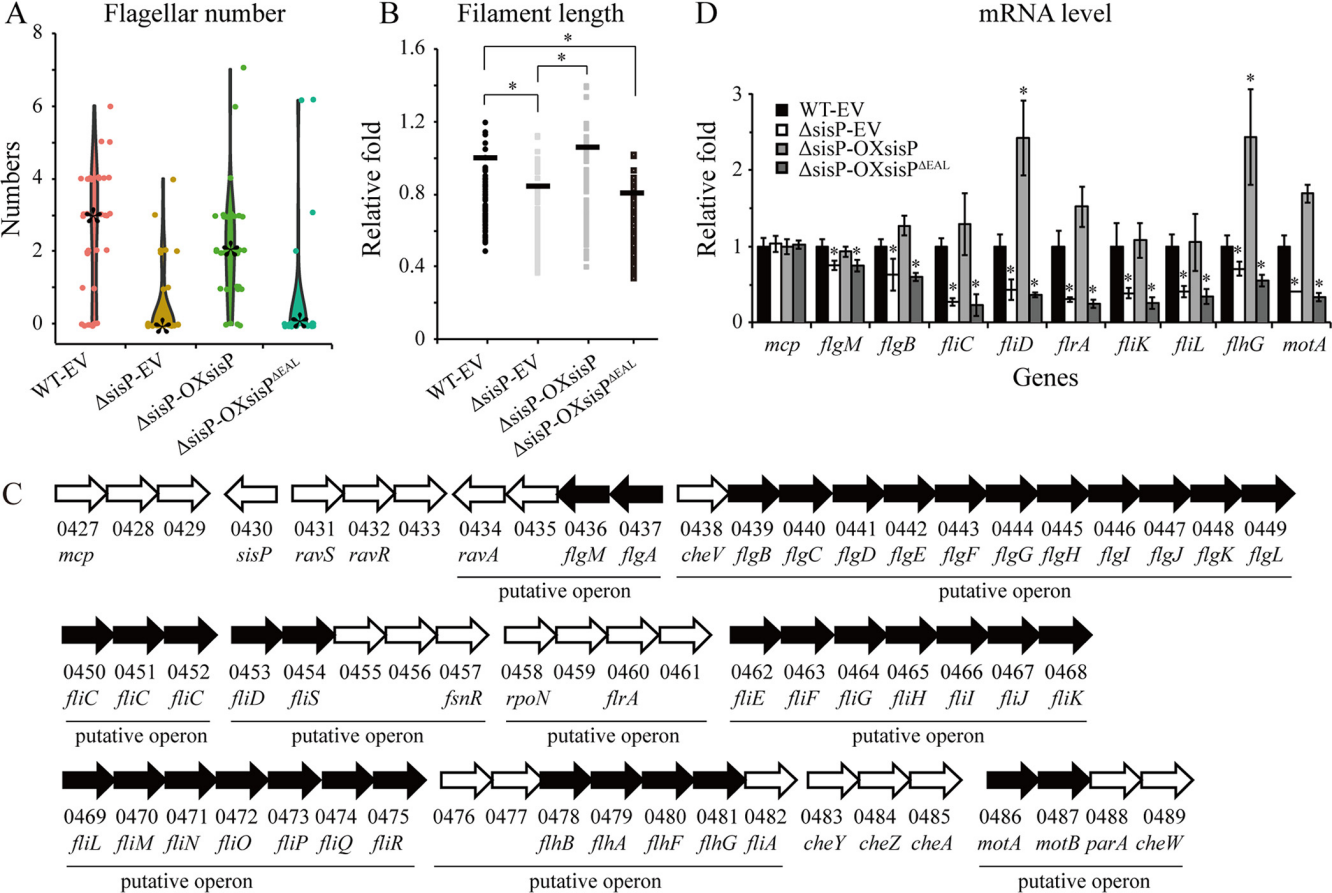
SisP positively regulates bacterial flagellar gene expression, filament length, and number of flagella in S. maltophilia. (A) Counting of flagella on >80 cells from each strain. *, Median values. (B) Length measurement of 60 bacterial filaments and calculation of fold changes relative to the average filament length in the WT strain bearing the EV. Horizontal lines indicate average filament lengths. (C) Schematic representation of the *sisP* gene locus with many flagellar genes located nearby. Arrows indicate coding sequences (CDSs) and the direction of transcribed. Black arrows indicate flagellar genes. Predicated operons are indicated. (D) Transcript levels of the indicated genes measured using qRT-PCR with tmRNA as the reference and calculation of the fold changes relative to transcript levels in the WT strain. Data represent at least three biological repeats. Error bars represent standard deviations. Student’s *t* tests were performed (*, *P* ≤ 0.05).

To further explore the regulatory role of *sis*P in modulating swimming motility, we measured the numbers and lengths of bacterial flagella, which are essential for bacterial motility. We found that most of the cells of the ΔsisP-EV and ΔsisP-OXsisP^ΔEAL^ strains had no flagella, while most of the cells of the WT-EV and ΔsisP-OXsisP strains had two to four flagella (Fig. 4A), suggesting that SisP plays a critical role in the synthesis and assembly of bacterial flagella through its PDE activity. In addition, by measuring the filament lengths of all strains that exhibited at least one flagellum, we found that deletion of *sisP* or the sequences encoding the EAL domain (the ΔsisP-EV- and ΔsisP-OXsisP^ΔEAL^ strains) significantly decreased the average length of bacterial filaments to 0.82- and 0.81-fold that of the WT-EV strain, respectively (Fig. 4B), demonstrating that SisP also positively regulates flagellar filament length. Considering that *sisP* is located adjacent to genes involved in signal transduction regulation, chemotaxis, and flagellar synthesis (Fig. 4C), we speculated that SisP may facilitate flagellar synthesis and assembly by positively regulating flagellar gene expression. As expected, quantitative reverse transcription-PCR (qRT-PCR) analysis verified that deletion of *sisP* or the EAL domain encoding sequences specifically and significantly reduced the transcriptional levels of the nine *flrA* genes belonging to class 1 of the flagellar gene transcription hierarchy; the *fliK*, *fliL*, and *flhG* genes belonging to class 2; the *flgB* and *fliC* genes belonging to class 3; and the *flgM*, *motA*, and *fliD* genes belonging to class 4, each of which was randomly chosen from each of the nine operons shown in Fig. 4C (Fig. 4D, the ΔsisP-EV and ΔsisP-OXsisP^ΔEAL^ strains). Therefore, SisP degrades c-di-GMP to facilitate flagellar synthesis and assembly through its positive regulation of flagellar gene expression.

### SisP boosts the activity of FsnR by releasing it from the FsnR-c-di-GMP complex to increase flagellar gene expression.

**FIG 5:**
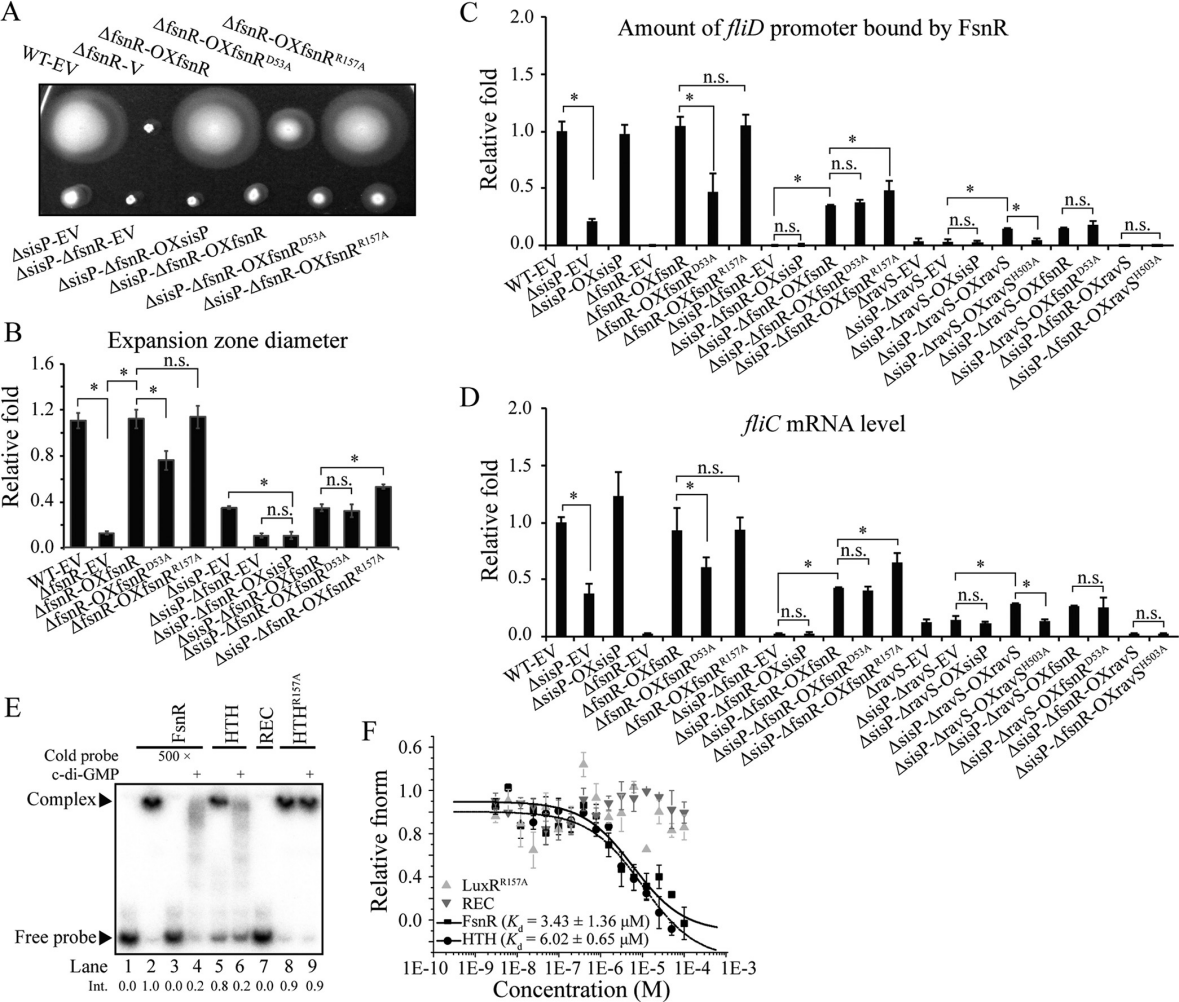
SisP stimulation of FsnR via release of FsnR from the FsnR-c-di-GMP complex. (A) Swimming motility in the following strains, demonstrating that regulation by *fsnR* acts downstream of *sisP*: genomic *fsnR* deletion bearing the EV pBBR1MCS2 (ΔfsnR-EV); constitutive expression of *fsnR*, *fsnR^D53A^*, and *fsnR^R157A^* by the recombinant plasmids pBBR1MCS2::*fsnR*, pBBR1MCS2::*fsnR^D53A^*, and pBBR1MCS2::*fsnR^R157A^* in the background of genomic *fsnR* deletion (ΔfsnR-OXfsnR, ΔfsnR-OXfsnR^D53A^, and ΔfsnR-OXfsnR^R157A^, respectively); genomic *fsnR* and *sisP* deletions bearing pBBR1MCS2 (ΔsisP-ΔfsnR-EV); and constitutive expression of *sisP*, *fsnR*, *fsnR^D53A^*, and *fsnR^R157A^* in the background of genomic *sisP* and *fsnR* deletions (ΔsisP-ΔfsnR-OXsisP, ΔsisP-ΔfsnR-OXfsnR, ΔsisP-ΔfsnR-OXfsnR^D53A^, and ΔsisP-ΔfsnR-OXfsnR^R157A^, respectively). (B) Measurement and quantification of the expansion zone diameters corresponding to panel A and calculation of the fold changes relative to the diameters in the WT strain. (C) Positive regulation by *sisP* of FsnR binding affinity to the *fliD* promoter shown by chromatin immunoprecipitation and quantitative PCR: measurement of *fliD* promoter binding by FsnR using FsnR polyclonal antibodies to capture the FsnR-*fliD* promoter complex, and calculation of fold changes relative to binding in the WT-EV strain. (D) SisP positively regulates activity of FsnR in increasing transcription of *fliC*. The amount of *fliC* mRNA was measured by qRT-PCR with tmRNA as the internal control. (E) EMSA of FsnR-*fliD* promoter complexes, demonstrating that c-di-GMP inhibits the binding affinity of FsnR for the *fliD* promoter through its HTH domain, with Arg^157^ as the essential site. The same amount of labeled DNA was added into each reaction; unlabeled DNA (a 500-fold higher concentration than labeled DNA) was used as the competitor for binding FsnR; 10 μM c-di-GMP was added into the reaction system as indicated. Free probe = unbound, labeled *fliD* promoter DNA. “Int.” represents the band intensity of the complexes formed by the labeled DNA probe and the indicated protein. The lane number and the Int. are indicated below the panel. (F) MST assay measurements of interactions between the indicated proteins and c-di-GMP, demonstrating that FsnR binds directly to c-di-GMP through its HTH domain, with Arg^157^ as the essential site. The *K_d_* was quantified to estimate the binding affinity of direct interactions. Data represent at least three biological repeats. Error bars represent standard deviations. Student’s *t* tests were performed (*, *P* ≤ 0.05; n.s., no significant differences).

Next, we investigated the regulatory mechanisms employed by SisP to regulate flagellar gene expression. We noticed that all of the flagellar genes regulated by SisP are also regulated by FsnR (23). We speculated that FsnR acts downstream of SisP in the regulation of flagellar gene expression and used genetic analyses to verify this hypothesis. Consistent with the reported results, deletion of *fsnR* (the ΔfsnR-EV strain) abolished bacterial swimming motility and remarkably reduced the transcriptional level of *fliC*, while constitutive expression of *fsnR* in the background of the genomic *fsnR* deletion (the ΔfsnR-OXfsnR strain) completely restored the WT pattern (the WT-EV strain) (Fig. 5A, B, and D). Also, FsnR directly binds to the *fliD* promoter for the appearance of FsnR-*fliD* promoter complexes in the electrophoretic mobility shift assay (EMSA) (Fig. 5E, lane 2) and the capture of the *fliD* promoter using the polyclonal antibody of FsnR (Fig. 5C, the WT-EV and ΔfsnR-OXfsnR strains compared to the ΔfsnR-EV strain). Next, we used epistatic analysis to investigate the putative relationship between *sisP* and *fsnR* in the regulation of flagellar gene expression. As expected, deletion of both *sisP* and *fsnR* (the ΔsisP-ΔfsnR-EV strain) abolished bacterial swimming and significantly decreased the amounts of *fliD* promoter captured by the polyclonal antibody of FsnR and remarkably reduced the transcriptional level of *fliC*, one of the target genes regulated by SisP (Fig. 4D), similar to the deletion of *fsnR* (the ΔfsnR-EV strain) (Fig. 5A to D), suggesting that *fsnR* acts downstream of *sisP* in controlling flagellar gene expression and bacterial swimming motility. However, constitutive expression of *fsnR* (the ΔsisP-ΔfsnR-OXfsnR strain), not *sisP* (the ΔsisP-ΔfsnR-OXsisP strain), in a ΔsisP-ΔfsnR background (i.e., the ΔsisP-ΔfsnR-EV strain) partially and significantly restored its phenotypes, in aspects of bacterial swimming motility, the amounts of *fliD* promoter bound by FsnR, and the transcriptional level of *fliC* (Fig. 5A to D). This led us to speculate that SisP regulates the activity of FsnR rather than its expression level. Alternatively, SisP has more than one target in regulating bacterial swimming motility.

Next, we investigated the regulatory mechanism by which SisP modulates the activity of FsnR. Because SisP hydrolyzes c-di-GMP, which usually directly binds and modulates activities of its effectors, we speculated that FsnR may be an as-yet-unidentified c-di-GMP effector. Using EMSA, we found that the addition of c-di-GMP into the reaction system remarkably reduced the formation of FsnR-*fliD* promoter complexes (Fig. 5E, lane 4), which is suggestive of the direct inhibition of FsnR promoter-binding activity by c-di-GMP. In addition, MST analysis showed that c-di-GMP directly bound to FsnR with a moderate binding affinity (*K_d_* = 3.43 ± 1.36 μM, larger than 0.5 μM) (Fig. 5F) (38). These results confirmed the identification of FsnR as a newly discovered c-di-GMP effector whose activity is inhibited by c-di-GMP binding.

The FsnR protein has two domains: REC is the conserved domain of an RR, and HTH is a helix-turn-helix DNA-binding domain. Both of these domains were separately analyzed by EMSA to examine their ability to bind the *fliD* promoter. As expected, only HTH directly bound the *fliD* promoter, as exhibited by the formation of HTH-*fliD* promoter complexes, but not REC-*fliD* promoter complexes (Fig. 5E, lanes 5 and 7). Moreover, HTH-*fliD* promoter complexes disappeared after the addition of c-di-GMP (lane 6), suggesting that it might be the HTH domain of FsnR that directly binds to c-di-GMP. Further MST analyses using the two separate domain proteins of FsnR were undertaken to verify this speculation. As expected, the HTH domain protein bound directly to c-di-GMP with similar affinity (*K_d_* = 6.02 ± 0.65 μM) to that of the full-length FsnR binding to c-di-GMP, and there was no detectable interaction between the REC domain protein and c-di-GMP (Fig. 5F). Next, we constructed a putative FsnR-c-di-GMP complex through molecular docking analysis, which illustrated that c-di-GMP might form hydrogen bonds with Arg^153^, Gln^154^, Arg^157^, and Arg^158^ (see Fig. S5A). However, amino acid sequence alignment of FsnR and its homologs within *Stenotrophomonas* showed that only Arg^157^ is conserved (see Fig. S5B), which suggests an essential role of Arg^157^ in the FsnR-c-di-GMP interaction. We then constructed an HTH domain protein with Arg^157^ mutated to Ala (protein HTH^R157A^) and investigated its interaction with c-di-GMP. As expected, no direct interaction was detected in the MST assay (Fig. 5F), which was consistent with results of the EMSA showing that HTH^R157A^ was blind to c-di-GMP in binding to the *fliD* promoter (Fig. 5E). Therefore, c-di-GMP appears to inhibit the activity of FsnR though direct binding of the HTH domain in a manner requiring the Arg^157^ residue and independent of the REC domain. These results suggested that the activity of FsnR can be boosted by degradation of its interaction partner c-di-GMP, which we speculated might be carried out by SisP.

10.1128/mbio.01414-22.6 FIG S5 Putative essential role of Arg^157^ in the interaction between S. maltophilia FsnR and c-di-GMP. (A) Predicted docking site between FsnR and c-di-GMP. Blue lines indicate potential hydrogen bonds. (B) BlastP sequence alignment showing conservation of Arg^157^ in *Stenotrophomonas*. Homologs of FsnR used in the alignment are from strains indicated on the left side. Four putative amino acid residues involved in hydrogen bond formation with c-di-GMP are shown in red in the FsnR sequence. In the sequences of the homologs, red indicates conserved sites, and green indicates nonconserved sites. Download FIG S5, TIF file, 1.9 MB. Copyright © 2022 Zhang et al. 2022 Zhang et al. https://creativecommons.org/licenses/by/4.0/ This content is distributed under the terms of the Creative Commons Attribution 4.0 International license.

If our hypothesis was correct, mutation of Arg^157^ to Ala should mimic SisP-mediated activation of FsnR and restore FsnR-mediated flagellar gene expression and swimming motility in a Δ*sisP* mutant. As expected, the site mutation of Arg^157^ to Ala significantly increased FsnR activity in terms of *fliD* promoter interaction, regulation of *fliC* expression, and facilitation of the swimming motility in the absence of SisP (Fig. 5A to D, the ΔsisP-ΔfsnR-OXfsnR^R157A^ strain compared to the ΔsisP-ΔfsnR-OXfsnR strain). This mutation had no effect on FsnR activity when SisP was present (the ΔfsnR-OXfsnR^R157A^ strain compared to the ΔfsnR-OXfsnR strain), presumably because under these conditions c-di-GMP levels are low enough to activate FsnR. These results suggested that the site mutation of Arg^157^ to Ala mimics the activation of FsnR by SisP.

In summary, our results thus far indicated that the transcription factor FsnR is a newly discovered c-di-GMP effector with an unreported c-di-GMP binding domain, HTH, and that Arg^157^ within the HTH domain plays an essential role in its interaction with c-di-GMP. Through binding with c-di-GMP, FsnR’s role in evoking flagellar gene transcription is suppressed. SisP, by degrading c-di-GMP, acts to release FsnR from the c-di-GMP-FsnR complex and stimulate FsnR. However, the site mutation of Arg^157^ to Ala significantly increased, yet only partially rescued, the transcriptional activity of FsnR in the absence of SisP (Fig. 5A to D, the ΔsisP-ΔfsnR-OXfsnR^R157A^ strain compared to the WT-EV strain), indicating that an additional strategy is employed by SisP to regulate FsnR activity and that both are required for full activation of FsnR.

### SisP elevates the phosphorylation level of FsnR to stimulate its activity.

Besides being a transcription factor, FsnR is an RR with a conserved REC domain. Because the activity of an RR is related to its phosphorylation level, we speculated that a second regulatory strategy employed by SisP to regulate FsnR activity might be modulation of the FsnR phosphorylation level. Thus, we investigated whether the phosphorylation level of FsnR plays a role in the regulation of its activity. Mutation of the conserved phosphorylation site Asp^53^ to Ala in the REC domain of FsnR remarkably diminished FsnR activity (Fig. 5A to D, the ΔfsnR-OXfsnR strain compared to the ΔfsnR-OXfsnR^D53A^ strain), suggesting that the phosphorylation level of FsnR is positively correlated with its activity. This change in FsnR activity was depleted in the absence of SisP (the ΔsisP-ΔfsnR-OXfsnR strain compared to the ΔsisP-ΔfsnR-OXfsnR^D53A^ strain), indicating that the phosphorylation level of FsnR is under the control of SisP and that the Asp^53^ to Ala mutation within the REC domain does not affect the c-di-GMP binding affinity and inhibition of FsnR. Therefore, the alternative strategy used by SisP to stimulate FsnR may involve the positive regulation of FsnR phosphorylation level, whose effect may not be mimicked by a heterologously produced PDE for the similar swimming motility of ΔsisP-EV and ΔsisP-OXpcrK strains (Fig. 2A to C).

In addition, we attempted to mimic constitutive phosphorylation of FsnR in the absence of SisP by replacing the conserved Asp^53^ with Glu, Arg, and Lys, respectively (the ΔsisP-ΔfsnR-OXfsnR^D53E^, ΔsisP-ΔfsnR-OXfsnR^D53R^, and ΔsisP-ΔfsnR-OXfsnR^D53K^ strains), because these substitutions have worked for some RR proteins (37). However, there was no improvement in bacterial swimming motility in these strains compared to the ΔsisP-ΔfsnR-OXfsnR^D53A^ strain (see Fig. S6), suggesting that our efforts at mimicking constitutive phosphorylation of FsnR failed. Because the strategy to mimic the constitutive phosphorylation of a given RR might be so distinctive, the replacement of Asp^53^ by some other amino acid residue should be tried in order to mimic the constitutive phosphorylation of FsnR.

10.1128/mbio.01414-22.7 FIG S6 Substitution of FsnR Asp^53^ fails to mimic the constitutively phosphorylated state of FsnR. Swimming motility of S. maltophilia strains with constitutive expression of *fsnR fsnR^D53A^*, *fsnR^D53E^*, *fsnR^D53K^*, and *fsnR^D53R^*, in the background of genomic *sisP* and *fsnR* deletions (ΔsisP-ΔfsnR-OXfsnR, ΔsisP-ΔfsnR-OXfsnR^D53A^, ΔsisP-ΔfsnR-OXfsnR^D53E^, ΔsisP-ΔfsnR-OxfsnR^D53K^, and ΔsisP-ΔfsnR-OXfsnR^D53R^, respectively), with WE-EV as a positive control control and ΔsisP-ΔfsnR-EV as a negative control. Data represent three biological repeats. Download FIG S6, TIF file, 1.5 MB. Copyright © 2022 Zhang et al. 2022 Zhang et al. https://creativecommons.org/licenses/by/4.0/ This content is distributed under the terms of the Creative Commons Attribution 4.0 International license.

**FIG 6:**
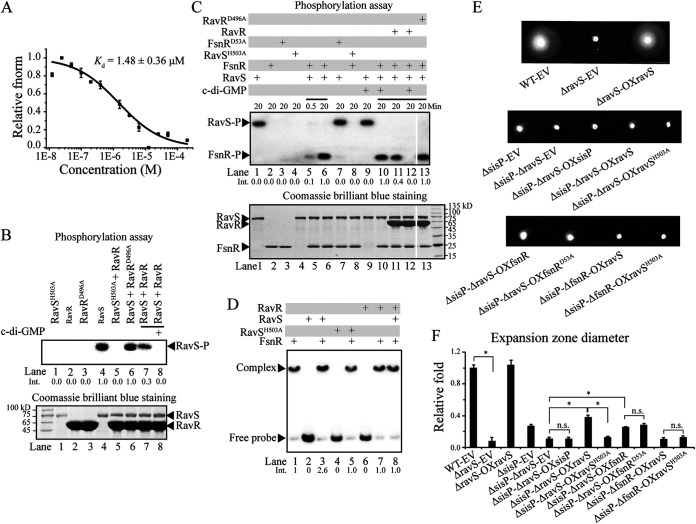
SisP stimulates FsnR via elevation of its phosphorylation level. (A) MST measurements and quantification of the *K_d_* of the direct interaction between RavS and c-di-GMP using 20 nM fluorescein-labeled RavS protein and increasing concentrations of c-di-GMP. (B, upper panel) Autoradiography of phosphorylation bands on SDS-PAGE of an *in vitro* phosphotransfer assay used to examine the putative phosphotransfer from RavS to RavR facilitated by c-di-GMP. RavS^H503A^ and RavR^D496A^ were used as negative controls. RavS was incubated with [γ-^32^P]ATP with or without 10 μM c-di-GMP for 30 min. (Lower panel) The same gel stained with Coomassie brilliant blue after autoradiography to check the amounts of each protein. “Int.” represents the intensity of the RavS-P measured by ImageJ software. The lane number and Int. are indicated below the panel. (C) c-di-GMP decreases the phosphorylation level of FsnR by promoting competition of RavR in grabbing the phosphoryl group of RavS. (Upper panel) *In vitro* phosphotransfer assay to examine the effects of RavS, RavR, and c-di-GMP on the regulation of the FsnR phosphorylation level. Purified RavS was incubated with RavR, or FsnR, or both proteins as indicated in the presence of [γ-^32^P]ATP and 10 μM c-di-GMP, as indicated for the times indicated above the panel. “Int.” represents the intensity of the band of the phosphorylated FsnR (FsnR-P) measured by ImageJ software. The lane number and Int. are indicated below the panel. The picture shown is the image of the same gel. (Lower panel) The same gel stained with Coomassie brilliant blue to check amounts of the proteins after autoradiography. The lane number is below the panel. (D) Phosphorylation of FsnR by RavS to promote formation of the FsnR-*fliD* promoter complexes, with RavR as an antagonist, as shown by EMSAs of FsnR protein preincubated with RavS, RavS^H503A^, and RavR, as well as [γ-^32^P]ATP, as indicated, in phosphorylation buffer for 20 min. The lane number and Int. are indicated below the panel. (E) Swimming motility of the following strains, showing SisP-mediated elevation of the phosphorylation level of FsnR via RavS: genomic *ravS* deletion bearing the EV pBBR1MCS2 (ΔravS-EV); constitutive expression of *ravS* by the recombinant plasmid pBBR1MCS2::*ravS* in the background of genomic *ravS* deletion (ΔravS-OXravS); genomic *sisP* and *ravS* deletions bearing pBBR1MCS2 (ΔsisP-ΔravS-EV); constitutive expression of *sisP*, *ravS*, *ravS^H503A^*, *fsnR*, and *fsnR^D53A^* in the background of genomic *sisP* and *fsnR* deletions (ΔsisP-ΔravS-OXsisP, ΔsisP-ΔravS-OXravS, ΔsisP-ΔravS-OXravS^H503A^, ΔsisP-ΔravS-OXfsnR, and ΔsisP-ΔravS-OXfsnR^D53A^, respectively); and constitutive expression of *ravS* and *ravS^H503A^* in the background of genomic *sisP* and *fsnR* deletions (ΔsisP-ΔfsnR-OXravS and ΔsisP-ΔfsnR-OXravS^H503A^, respectively). (F) Measurement and quantification of the expansion zone diameters corresponding to panel E and calculation of the fold changes relative to the diameters in the WT strain. Data represent at least three biological repeats. Error bars represent standard deviations. Student’s *t* tests were performed (*, *P* ≤ 0.05; n.s., no significant differences).

How might SisP positively regulate the phosphorylation level of FsnR? Because FsnR is directly phosphorylated by an unknown cognate HK, it is plausible that SisP promotes the phosphotransfer from the HK to FsnR. Because clustered genes in prokaryotic genomes have similar or related functions, we speculated that the HK RavS, which is encoded by *ravS* located in the operon transcribed in the opposite direction as the single *sisP* gene (Fig. 4C), might be the cognate HK of FsnR. We performed *in vitro* phosphotransfer assays to verify whether RavS could transfer the phosphoryl group to FsnR. As shown in Fig. 6C, purified RavS, but not RavS^H503A^ (conserved His^503^ substituted by Ala), autophosphorylated in the presence of ATP. Furthermore, within 30 s, phosphorylated RavS transferred its own phosphoryl group to FsnR, but not to FsnR^D53A^, whose conserved phosphorylation site Asp^53^ was mutated to Ala (Fig. 6C, lanes 1 to 8). Given the consensus that an HK transfers the phosphoryl group to its paired RR within 1 min, this result indicated that RavS and FsnR constitute a two-component signal transduction system. In addition, as expected, more FsnR-*fliD* promoter complexes were formed *in vitro* in the presence of phosphorylated RavS than unphosphorylated RavS^H503A^ (Fig. 6D, lanes 3 and 5), demonstrating that RavS transfers the phosphoryl group to FsnR to stimulate its DNA binding activity.

As previously reported, c-di-GMP directly binds RavS^Xcc^ (the homolog of RavS in X. campestris) (20), and Arg^656^, which is essential for the RavS^Xcc^-c-di-GMP interaction was shown to be conserved in the RavS encoded by S. maltophilia (see Fig. S7). We speculated that c-di-GMP directly binds RavS and regulates its activity or phosphotransfer to FsnR, thereby modulating the phosphorylation levels of RavS and FsnR. When we performed the MST assay to test for a direct interaction between RavS and c-di-GMP, we found that c-di-GMP binds to RavS with a moderate binding affinity (*K_d_* = 1.48 ± 0.36 μM, which is larger than 0.5 μM) (Fig. 6A) (38). Next, we carried out *in vitro* autophosphorylation and phosphotransfer assays to investigate whether the interaction with c-di-GMP regulates the phosphorylation levels of RavS and FsnR. No differences in the phosphorylation levels of RavS or FsnR were detected following the addition of c-di-GMP (Fig. 6C, lanes 9 and 10 compared to lanes 1 and 6), suggesting that c-di-GMP regulates neither the autokinase activity of RavS nor the phosphotransfer from RavS to FsnR.

10.1128/mbio.01414-22.8 FIG S7 RavS-RavR is conserved in S. maltophilia and X. campestris pv. *campestris*. BlastP sequence alignment was performed between RavS and RavS^Xcc^ and between RavR and RavR^Xcc^. Protein domains were predicted by SMART, and their names are indicated below. The conserved Arg residue is indicated with a five-pointed star. Download FIG S7, TIF file, 1.6 MB. Copyright © 2022 Zhang et al. 2022 Zhang et al. https://creativecommons.org/licenses/by/4.0/ This content is distributed under the terms of the Creative Commons Attribution 4.0 International license.

Previous studies in X. campestris showed that RavS^Xcc^ phosphorylates the RR RavR^Xcc^ (the homolog of S. maltophilia RavR) and that c-di-GMP binds RavS^Xcc^ to promote its phosphotransfer to RavR^Xcc^ (20). Sequence alignment showed relatively high conservation between RavS-RavR and RavS^Xcc^-RavR^Xcc^: 96% coverage and 55.57% identity between RavS and RavS^Xcc^ and 99% coverage and 83.13% identity between RavR and RavR^Xcc^ (see Fig. S7). Thus, we speculated the following: RavS transfers the phosphoryl group to RavR in S. maltophilia, RavR competes with FsnR for the phosphoryl group of RavS, and c-di-GMP indirectly decreases the phosphorylation level of FsnR by promoting the phosphotransfer from RavS to RavR. The following experiments were designed to verify these speculations. As shown in Fig. 6B, a significant decrease in the RavS phosphorylation level was detected when RavR, but not RavR^D496A^ (conserved phosphorylation site Asp^496^ substituted by Ala), was added to the reaction system. A further decrease was detected when c-di-GMP was added together with RavR (lanes 6 to 8 compared to lane 4), suggesting that RavS transfers the phosphoryl group to RavR and that this process is promoted by c-di-GMP, which is consistent with the findings in X. campestris. Similar to RavR^Xcc^, no band corresponding to phosphorylated RavR were detected, probably because of its short half-life or quick dephosphorylation. Furthermore, there was a significant decrease in FsnR phosphorylation level when RavR, instead of RavR^D496A^ was added to the RavS-FsnR reaction system, and a further decrease was detected when c-di-GMP was added together with RavR (Fig. 6C, lanes 11 to 13 compared to lane 6). These results indicated that RavR competes with FsnR for the phosphoryl group of RavS and that c-di-GMP significantly increases the RavS-RavR phosphotransfer, leading to less RavS-FsnR phosphotransfer and a lower phosphorylation level of FsnR. Therefore, c-di-GMP appears to indirectly decrease the phosphorylation level of FsnR through binding RavS and promoting its phosphotransfer to RavR, leading to the speculation that SisP acts upstream to degrade c-di-GMP binding with RavS.

Genetic analyses to verify this speculation are shown in Fig. 6E and F and Fig. 5C and D. The ΔsisP-ΔravS-EV strain (deletions of genomic *sisP* and *ravS*) was similar to the ΔravS-EV strain, but not the ΔsisP-EV strain, with regard to FsnR binding affinity to the *fliD* promoter, the transcriptional level of *fliC*, and the bacterial swimming motility, suggesting that RavS acts downstream of SisP in the regulation of FsnR activity. Furthermore, as expected, constitutive expression of RavS, but not RavS^H503A^, in the ΔsisP-ΔravS background partially rescued FsnR activity (the ΔsisP-ΔravS-OXravS and ΔsisP-ΔravS-OXravS^H503A^ strains compared to the ΔsisP-ΔravS-EV strain). In contrast, there was no restoration of RavS-mediated regulation of swimming motility in the absence of *fsnR* (the ΔsisP-ΔfsnR-OXravS and ΔsisP-ΔfsnR-OXravS^H503A^ strains compared to the ΔsisP-ΔfsnR-EV strain), and no difference on the activities of FsnR and FsnR^D53A^ was identified in the absence of *sisP* and *ravS* (the ΔsisP-ΔravS-OXfsnR and ΔsisP-ΔravS-OXfsnR^D53A^ strains compared to the ΔsisP-ΔravS-EV strain) in regulating flagellar gene expression and bacterial swimming motility. These results indicated that SisP promotes the phosphorylation level of FsnR through its regulation of RavS phosphorylation level. Therefore, we uncovered an alternative regulatory strategy employed by SisP wherein SisP degrades c-di-GMP to eliminate its promotion of the phosphotransfer from RavS to RavR, indirectly increasing RavS-FsnR phosphotransfer and leading to a higher phosphorylation level of FsnR, which is the more active form of FsnR in eliciting flagellar gene expression.

## DISCUSSION

**FIG 7:**
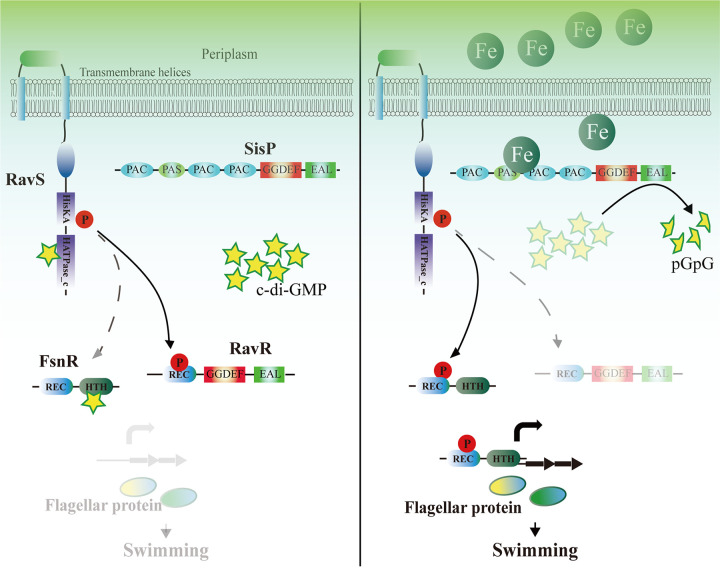
Model of SisP detection of ferrous iron to stimulate bacterial swimming motility. (Left panel) c-Di-GMP directly binds FsnR. Simultaneously, c-di-GMP decreases FsnR phosphorylation level by binding RavS to promote the phosphoryl transfer to RavR, which results in less RavS-FsnR phosphoryl transfer. Both actions are required for inhibition of the activity of FsnR in facilitating flagellar gene expression. (Right panel) Ferrous iron stimulates the PDE activity of SisP through direct binding to its PAC-PAS-PAC-PAC sensor domains. The activated SisP degrades c-di-GMP to release the direct inhibition of FsnR activity by c-di-GMP and to elevate FsnR phosphorylation level by release of the promotion of c-di-GMP in RavS-RavR phosphoryl transfer. Also, both are required for the full activation of FsnR in upregulating flagellar gene expression, thereby facilitating bacterial swimming motility. Red circles with P indicate phosphoryl groups, green circles with Fe indicate ferrous iron, and partially transparent and dashed lines indicate weakened and inactive reactions, respectively.

The results of this study led us to decipher a “one stone, two birds” strategy of c-di-GMP in regulating bacterial flagellar gene transcription (Fig. 7). We found that c-di-GMP binds the transcription factor FsnR directly, inhibiting its binding affinity for the promoters of flagellar genes, and simultaneously binds the HK RavS, promoting its phosphotransfer to RavR, which leads to less RavS-FsnR phosphotransfer and the lower phosphorylation level of FsnR. These two actions of c-di-GMP have additive effects on FsnR that disable its ability to elicit flagellar gene transcription, thereby diminishing bacterial swimming motility. However, this dual inhibition of FsnR activity by c-di-GMP can be eliminated by the phosphodiesterase SisP. Upon stimulation with ferrous iron, SisP degrades c-di-GMP, which releases RavS and FsnR from their complexes with c-di-GMP, resulting in a higher FsnR phosphorylation level and more FsnR unbound by c-di-GMP, both of which are required for the full activation of FsnR as a flagellar gene transcription factor. Once the flagellar proteins have been synthesized and assembled, the bacteria can swim.

Extensive research efforts have led to the identification of signals that stimulate c-di-GMP turnover. Many signals have been shown to modulate the activities of c-di-GMP turnover enzymes through intermediate receptors. However, most c-di-GMP turnover enzymes have sensor domains, including PAS (Per-Arnt-Sim), GAF (mammalian cGMP-regulated PDEs, *Anabaena* adenylyl cyclases, and E. coli transcription activator FhlA), and CHASE (cyclases/histidine kinases associated sensing extracellular), suggestive of their direct perception of stimuli and the putative regulation of their activities by antagonists or activators (33, 39, 40). Researchers have sought to identify direct signals recognized by the sensor domains of these enzymes and to dissect the underlying mechanisms. Some progress has been made. For instance, oxygen specifically binds to the globin domain and increases the cyclase activity of globin domain-containing DGCs from Escherichia coli, Bordetella pertussis, and Desulfotalea psychrophila (41). Light, via the BLUF domain, activates the phosphodiesterase activity of BlrP1 from Klebsiella pneumoniae (42). However, the specific signals directly perceived by most DGCs and PDEs with sensor domains remain unknown. In this study, we identified ferrous iron as the specific signal perceived by SisP. While iron is a critical nutrient required for the basic metabolic processes of human cells, ferrous iron is an elicitor of the production of reactive oxygen species that damage important macromolecules and lead to lipid peroxidation, endothelial injury, protein oxidation, mitochondrial injury, and DNA damage (43). Human physiologic systems have evolved to orchestrate a careful balance of iron binding and release that enables us to avoid injury while effectively delivering this essential nutrient to tissues. In acute illnesses, however, iron homeostasis is often disrupted and ferrous iron is produced, which contributes to tissue injury, as well as to a higher risk of bacterial infection (44, 45). Many investigations, based in clinical and laboratory settings, have shown that iron supplementation causes more severe infections of Mycobacterium tuberculosis (46) but lower virulence of the enteric pathogen *Citrobacter* (47). On the basis of these reports, iron appears to play pleiotropic roles, or even reversed roles, in different bacterial species. This study deciphered the role of ferrous iron in regulating the swimming motility of S. maltophilia. Ferrous iron acts as a specific signal, detected by SisP, to increase flagellar gene expression, thereby facilitating bacterial swimming motility. Therefore, the burst release of ferrous iron in humans with acute illnesses may serve as a signal for bacteria located at damage sites to swim inward or flee from the hostile environment, thereby contributing to the higher risk of bacterial infection or spread. If true, this phenomenon may provide a reference for the development of strategies to reduce S. maltophilia infection or spread.

Many c-di-GMP effectors, but few in comparison to c-di-GMP turnover enzymes, have been identified, which include transcription factors, HKs, RRs, riboswitches, and degenerated DGCs and PDEs (43, 44). Among the reported c-di-GMP binding HKs and RRs, distinct domains are used to interact with c-di-GMP. Among these HKs, the orphan HK SgmT from Myxococcus xanthus is bound by c-di-GMP via its GGDEF domain, leading to the inhibition on its autophosphorylation level (48). Two cell cycle related HKs, CckA and ShkA, from *Caulobacter* bind with c-di-GMP through the catalytic ATP-binding (CA) and the pseudo-REC domains, respectively, which switches CckA from the kinase to the phosphatase mode (49, 50) but activates the kinase activity of ShkA (51, 52). In this study and in a reported case in X. campestris (17), the HK RavS binds c-di-GMP through the CA domain to enhance the RavS-RavR phosphotransfer. Among the c-di-GMP binding RRs, both RRs from Vibrio cholerae, the REC-AAA+-HTH domain protein VpsR and the REC-HTH domain protein VpsT, via the AAA+ (ATPase associated with diverse cellular activities) and the REC domain, respectively, bind c-di-GMP to stimulate their transcriptional activities (53–55). In this study, FsnR binds c-di-GMP through the HTH domain to inhibit its activity. Therefore, it is speculated that HKs and RRs introduce c-di-GMP-sensitivity to the regulatory network might be general.

As reported in most cases, c-di-GMP behaves as an “on-off” switch to bind and modulate the activities of its effectors (56). Whether c-di-GMP has the flexibility to quantitatively regulate such activities remains to be answered. We found that c-di-GMP exerts two different types of regulation of FsnR activity: a molecular interaction and a covalent modification. This dual regulatory strategy provides flexible and additive control of FsnR activity, adding checkpoints to the regulation and providing a strategy to quantitatively modulate the activity of a c-di-GMP effector. Moreover, pGpG generated by SisP-mediated degradation of c-di-GMP might also bind and regulate FsnR, which should be studied in the future.

It was reported in a study of X. campestris that less RavS-RavR phosphotransfer and a higher phosphorylation level of RavS are required to drive bacterial swimming, but the exact regulatory mechanism remains unknown (17). Our work has elucidated the regulatory mechanism in S. maltophilia: less RavS-RavR phosphotransfer and a higher phosphorylation level of RavS elevates the phosphorylation level of FsnR through RavS-FsnR phosphotransfer, and phosphorylated FsnR is more active in eliciting flagellar gene expression, thereby facilitating swimming motility. Interestingly, we found that SisP degraded c-di-GMP to prevent it from binding with RavS, whereas the X. campestris study found that RavR^Xcc^ degraded c-di-GMP to prevent it from binding with RavS^Xcc^. Furthermore, insertional inactivation of genomic *ravR* depletes bacterial swimming motility in S. maltophilia (Fig. 1A and B), which is contrary to the finding that in-frame deletion of *ravR^Xcc^* significantly increases the swimming motility of X. campestris. These results necessitate further investigations to verify the role of RavR in S. maltophilia and to dissect the putative cross talk between RavR and SisP in the hydrolysis of c-di-GMP bound with RavS.

It is also interesting to find that the HTH domain of FsnR bound both c-di-GMP and DNA motifs of its target promoters, suggesting that the putative regions or residues involved in both of these interactions may overlap. However, mutation of the Arg^157^ to Ala (the recombinant protein HTH^R157A^) abolished the interaction of HTH and c-di-GMP but barely influenced the binding of HTH to the *fliD* promoter (Fig. 5C and E), demonstrating that Arg^157^ is essential for the HTH–c-di-GMP interaction, but not the HTH-*fliD* promoter interaction. Further experiments, such as crystallization of both the HTH–c-di-GMP and HTH-DNA motif complexes are still needed to investigate how the FsnR–c-di-GMP interaction affects FsnR-DNA motif binding and to what extent the sites of FsnR overlap in binding with c-di-GMP and DNA motif.

In addition, c-di-GMP plays an important role in regulating flagellar motility, which has not been studied in S. maltophilia. Many of the c-di-GMP effectors involved in the regulation of flagellar synthesis and motility, such as FlrA and YcgR, are conserved in S. maltophilia. FlrA is inhibited by c-di-GMP binding in facilitating flagellar gene expression (12–15), while YcgR binds c-di-GMP and acts on a flagellar motor protein to regulate flagellar motility (57). Therefore, further studies are needed to illustrate that to what extent roles of these c-di-GMP effectors are conserved in S. maltophilia.

## MATERIALS AND METHODS

### Bacterial strains, culture media, plasmids, and primers.

The bacterial strains and recombinant plasmids used in this study are listed in Table S1. The draft genome sequence of *S. maltophilia* CGMCC 1.1788 is available at http://www.ncbi.nlm.nih.gov/bioproject/876580. The primers used for plasmid constructions and PCR are listed in Table S2. S. maltophilia strains were grown at 28°C in rich Luria-Bertani (LB) medium (tryptone, 10 g L^−1^; yeast extract, 5 g L^−1^; NaCl, 10 g L^−1^), or LB medium pretreated with 5 μM DFO for 2 h, followed by supplementation with the indicated ions together with the bacteria. All recombinant vectors were prepared using the E. coli DH5α strain. Insertional inactivation mutants were constructed by homologous, single-crossover recombination using the suicide vector pK18mob. In-frame deletion mutants were constructed by homologous, double-crossover recombination using the suicide vector pK18mobsacB. Genetic complementary vectors were constructed by inserting full-length sequences of the corresponding genes (under the control of P*lac* promoter) into the broad-host-range vector pBBR1MCS2. All general molecular biology operations were conducted in accordance with standard protocols in molecular cloning (58). Site mutations were constructed using the Fast Mutagenesis System (Transgene Biotech, Beijing, China). Ampicillin (100 μg mL^−1^) or kanamycin (25 μg mL^−1^) was included in cultures wherever necessary. Transformation of S. maltophilia was performed in a Bio-Rad Pulser XCell (Bio-Rad, Hercules, CA) at 18 kV cm^−1^, 25 μF, and 200 Ω.

10.1128/mbio.01414-22.9 TABLE S1 Bacterial strains and plasmids used in this study. Download Table S1, DOCX file, 0.03 MB. Copyright © 2022 Zhang et al. 2022 Zhang et al. https://creativecommons.org/licenses/by/4.0/ This content is distributed under the terms of the Creative Commons Attribution 4.0 International license.

10.1128/mbio.01414-22.10 TABLE S2 Primers used in this study. Download Table S2, DOCX file, 0.02 MB. Copyright © 2022 Zhang et al. 2022 Zhang et al. https://creativecommons.org/licenses/by/4.0/ This content is distributed under the terms of the Creative Commons Attribution 4.0 International license.

### Bacterial swimming motility assays, signal screening assay, and measurement of filament length and flagella number.

LB semisoft (0.15% agar) agar motility plates, plates prepared with different molecules (for instance, ferrous iron, ferric iron, zinc iron, animal hormones, and amino acids), or plates pretreated with 5 μM DFO for 2 h, followed by supplementation with the indicated irons, wherever necessary, were used. Strains cultured overnight were adjusted to an optical density at 600 nm (OD_600_) of 0.4. An aliquot (2 μL) of the culture was transferred into the motility plates, followed by incubation at 28°C for 24 to 26 h. The plates were photographed, and the bacterial expansion zones were measured. Part of the margin of the bacterial expansion zone was picked and used to prepare samples for videos using the Olympus BX51, and images for measurements of the filament lengths and flagellum numbers were obtained with a JEM-1400 electron microscope (JEOL, Japan) at an operating voltage of 80 kV. The flagellar filament length was then measured using ImageJ software.

### Biofilm formation ability assay.

The crystal violet staining method was used to quantify the formation of biofilm, as described in our previous study (23). In brief, bacterial cultures of strains grown overnight in LB medium were adjusted to an OD_600_ of 0.4, diluted in LB medium in 96-well polystyrene plates, and grown at 28°C for 16 h without shaking. The bacterial OD_600_ was measured on a Tecan Infinite 200 Pro microplate reader. Before measurement of the biofilm amount, the wells were washed with water carefully and dyed with 0.1% crystal violet for 30 min, followed by careful washing with water and solubilization of the crystal violet using 95% ethanol for 40 min. The OD_590_ was measured, and the OD_590_/OD_600_ ratio was used to represent the biofilm formation ability.

### MIC measurement.

Overnight bacterial cultures with the same OD_600_ were grown in LB medium containing a series concentration of meropenem at 28°C for 36 h without shaking and then photographed.

### Growth rate measurement.

Bacterial cultures were grown overnight in LB medium, adjusted to an OD_600_ of 0.4, and then diluted in LB medium in 96-well plates for the measurement of growth curves using an automatic microbial growth curve analyzer at 28°C with constant shaking for the entire period of measurement to prevent biofilm formation.

### Protein expression, purification, and polyclonal antibody preparation.

All proteins were expressed with a C-terminal His_6_ tag using recombinant pET30a vector or a glutathione *S*-transferase (GST) tag using pGEX-6P-1 vector; these were then transformed into E. coli BL21(DE3). His_6_-tagged proteins were extracted and purified through affinity chromatography using Ni-NTA agarose beads as recommended by the manufacturer (Novagen). Washing buffer with a higher concentration of imidazole was used, if necessary, to remove the non-His_6_-tagged proteins. GST-tagged proteins were expressed and purified with GST Resin (TRANS) according to the *GST Gene Fusion System Handbook* (Amersham Biosciences). During the purification process, on-column cleavage to deplete the GST tag was performed as described in our previous study (28). The purified proteins were stored in storage buffer (50 mM Tris-HCl [pH 8.0], 0.5 mM EDTA, 50 mM NaCl, 5% glycerol) for further use. Polyclonal antibodies against FsnR and RNA polymerase α-subunit were prepared by immunizing rats and rabbits with approximately 1.5 and 6 mg of purified, soluble proteins, respectively.

### Western blot assay.

S. maltophilia cells grown in LB medium to an OD_600_ of 1.0 were used for total protein extraction. A Western blot assay was performed in accordance with a standard protocol (51). Immunoblots were probed as described in a previous report (59). RNA polymerase α-subunit was detected as a loading control.

### EMSAs.

Assays were performed as described in our previous study (19, 23). The probe, the promoter region of *fliD*, was amplified by PCR and labeled by [γ-^32^P]ATP using T4 polynucleotide kinase. The labeled probes and purified proteins were incubated in reaction buffer containing 10 mM Tris (pH 7.0), 50 mM KCl, 1 mM dithiothreitol (DTT), 2.5% glycerol, 5 μM MgCl_2_, 50 ng L^−1^ poly(dI:dC), 0.05% NP-40, and 10 mM EDTA for 50 min at room temperature. ATP used to phosphorylate RavS was depleted using a PD-Spintrap G-25 desalting column (GE, New York, NY) before the addition of FsnR. Reactions were stopped by the addition of loading buffer (0.25% bromophenol blue, 40% sucrose), and samples were subjected to polyacrylamide gel electrophoresis (PAGE) in a 5% native gel and then exposed to a phosphor screen (GE Healthcare) for detection of the autoradiographic signals by a Typhoon FLA7000 (GE Healthcare). Unlabeled probes were used as the cold probes in the competition experiments.

### qRT-PCR.

The mRNA levels of all indicated genes were measured by qRT-PCR, as described in our previous study (59, 60). Total RNA was extracted using TRIzol (Invitrogen, USA), and treated with RNase-free DNase I (Ambion, USA) to eliminate any DNA contamination of the total RNA samples. First-strand cDNA was generated using random primers (Promega, USA) by Superscript III reverse transcriptase (Invitrogen). The qRT-PCR was conducted using Maxima SYBR green (Fermentas, USA) in a DNA Engine Option 2 System (Bio-Rad), according to the manufacturer’s instructions. The transfer-mRNA (tmRNA) level was used as the internal control.

### Chromatin immunoprecipitation and quantitative PCR.

These experiments were performed as described in our previous study with minor modifications (59). Briefly, bacterial cultures grown to an OD_600_ of 0.4 were cross-linked with 1% formaldehyde for 20 min and then quenched with 0.5 M glycine for 5 min. For DNA preparation, bacterial cultures were harvested, washed twice with cold phosphate-buffered saline (PBS) buffer, and resuspended in 1 mL of lysis buffer (10 mM Tris [pH 8.0], 20% sucrose, 50 mM NaCl, 10 mM EDTA) for cell lysis by a Diagenode Bioruptor UCD-300 (Diagenode, Seraing, Belgium). Next, 4 mL of IP buffer (50 mM HEPES-KOH [pH 7.5], 150 mM NaCl, 1 mM EDTA, 1% Triton X-100, 0.1% sodium deoxycholate, 1 mM phenylmethylsulfonyl fluoride) was added, and an aliquot was used for chromatin fragmentation. Then, 100 μL of this aliquot was set aside as the control DNA. For immunoprecipitation, FsnR-DNA complexes were captured by using protein A-Sepharose and FsnR polyclonal antibody, washed with IP buffer and IP buffer containing 500 mM NaCl, and eluted with elution buffer (50 mM Tris [pH 7.5], 10 mM EDTA, 1% sodium dodecyl sulfate [SDS]). Precipitated DNA was purified by a PCR purification kit (Qiagen, Dusseldorf, Germany) after treatment with RNase A and proteinase K. The amounts of captured DNA were normalized to the control DNA.

### *In vitro* autophosphorylation and phosphoryl transfer assay.

These experiments were performed as described in our previous study (59, 60). Briefly, purified RavS or RavS^H503A^ was incubated with c-di-GMP or the purified proteins as indicated in the presence of 20 μM [γ-^32^P]ATP (Perkin-Elmer, USA) in reaction buffer (50 mM Tris-HCl [pH 7.8], 25 mM NaCl, 25 mM KCl, 5 mM MgCl_2_, 2 mM DTT) at 28°C for the indicated times. In RavS-RavR phosphoryl transfer assays, purified RavS or RavS^H503A^ were incubated with 20 μM [γ-^32^P]ATP (Perkin-Elmer, USA) in reaction buffer for the indicated times at 28°C. The remaining ATP was depleted by using a PD-Spintrap G-25desalting column (GE, New York, NY) before the next step. c-di-GMP, wherever necessary, was added to the reaction system simultaneously with the other proteins. The reactions were terminated by adding 5× SDS-PAGE loading buffer. Samples were loaded into the gels for PAGE and then exposed to a phosphor screen (GE Healthcare) to detect autoradiographic signals by a Typhoon FLA7000 (GE Healthcare).

### MST assay.

Interactions between proteins and molecules, including c-di-GMP and metal ions, were measured using a Monolith NT.115 device as described in our previous studies (17, 28, 60). Briefly, proteins used in the assays were purified and labeled by Monolith NT.115 protein labeling kit Red NHS (MicroScale Thermophoresis grade). The labeled proteins were incubated with the indicated molecules in PBS buffer, and the interactions were measured and analyzed by using Nano Temper Analysis Software, from which dissociation constants (*K_d_*) were calculated.

### PDE and DGC activity assay.

The experiments were performed as described in our previous study with modification (19, 60). For the DGC activity test, SisP and its recombinant proteins were incubated with [α-^32^P]GTP (GTP) (Perkin-Elmer) in reaction buffer (300 mM NaCl, 50 mM Tris-HCl [pH 7.5], 20 mM MgCl_2_, 2 mM DTT), respectively, at 28°C for the indicated times, with the reported DGC DncV from E. coli as the positive control. For the PDE activity investigation, ^32^P-labeled c-di-GMP was synthesized with DncV at 37°C for 4 h. The reaction was terminated by heating at 98°C for 10 min, and the precipitated protein was removed by centrifugation at 20,000 × *g* for 5 min. SisP was incubated with the synthesized ^32^P-labeled c-di-GMP and different kinds of metal ions, wherever necessary, in reaction buffer at 28°C for the indicated time. Degradation and generation of c-di-GMP was detected by TLC using cellulose PEI TLC plates and running buffer [1:1.5 (vol/vol) of saturated (NH_4_)_2_SO_4_ and 1.5 M KH_2_PO_4_ (pH 3.6)]. The running time was 1.5 to 2.5 h. After development, the plates were air-dried and exposed to a storage phosphor screen (GE Health-care), and then the autoradiographic signals were recorded on a Typhoon FLA700 (GE Healthcare).

### *In vivo* c-di-GMP quantification.

c-Di-GMP was extracted with some modifications based on a previous report (19, 60). Briefly, 21 mL of bacterial culture grown to an OD_600_ of 0.8 was used for c-di-GMP quantification. Different metal ions, wherever necessary, were added to culture samples grown in LB medium pretreated with 5 μM DFO for 2 h and incubated for 2 h at 28°C without shaking; the samples were then collected for c-di-GMP extraction. A 1-mL aliquot of each culture was serially diluted and grown on LB plates for 36 h, and the bacterial clones were numbered. Culture samples (20 mL) were centrifuged at 5,000 × *g* at 4°C for 10 min, 0.6 M HClO_4_ was added to the cell pellet, and denatured proteins were removed by centrifugation. The supernatant was neutralized to pH 6.0 with 5 M K_2_CO_3_, recentrifuged to remove the precipitated KClO_4_, and used for liquid chromatography-tandem mass spectrometry (LC-MS/MS) analyses. The amount of c-di-GMP in samples was calculated using a standard curve generated from pure c-di-GMP (Sigma-Aldrich, St. Louis, MO) suspended in ddH_2_O (Biolog, Germany). The c-di-GMP levels were normalized to bacterial cells and quantified according to the bacterial cellular volume.

### Molecular docking analyses.

Molecular docking analyses were performed as described in our previous study (17). Briefly, the three-dimensional structure of FsnR was generated by homology modeling methods using SWISS-MODEL (54–57). The known structure of 5hev was used for multiple template homology modeling, and docking calculations were carried out using Autodock 4.2.6 software. The lowest energy conformation was chosen for c-di-GMP and FsnR interaction analysis.

10.1128/mbio.01414-22.1 VIDEO S1 SisP positively regulates bacterial swimming motility. Motile behaviors of the bacterial strains WT-EV (A), ΔsisP-EV (B), ΔsisP-OXsisP (C), and ΔsisP-OXsisP^ΔEAL^ (D). The results are representative of three biological experiments. Download VIDEO S1, MOV file, 10.8 MB. Copyright © 2022 Zhang et al. 2022 Zhang et al. https://creativecommons.org/licenses/by/4.0/ This content is distributed under the terms of the Creative Commons Attribution 4.0 International license.
