# Supplementary figures and images for: Dual Regulatory Role Exerted by Cyclic Dimeric GMP To Control FsnR-Mediated Bacterial Swimming
Source: mBio. 2022 Sep 7;13(5):e01414-22. doi: 10.1128/mbio.01414-22 (PMC9600515; doi:10.1128/mbio.01414-22)

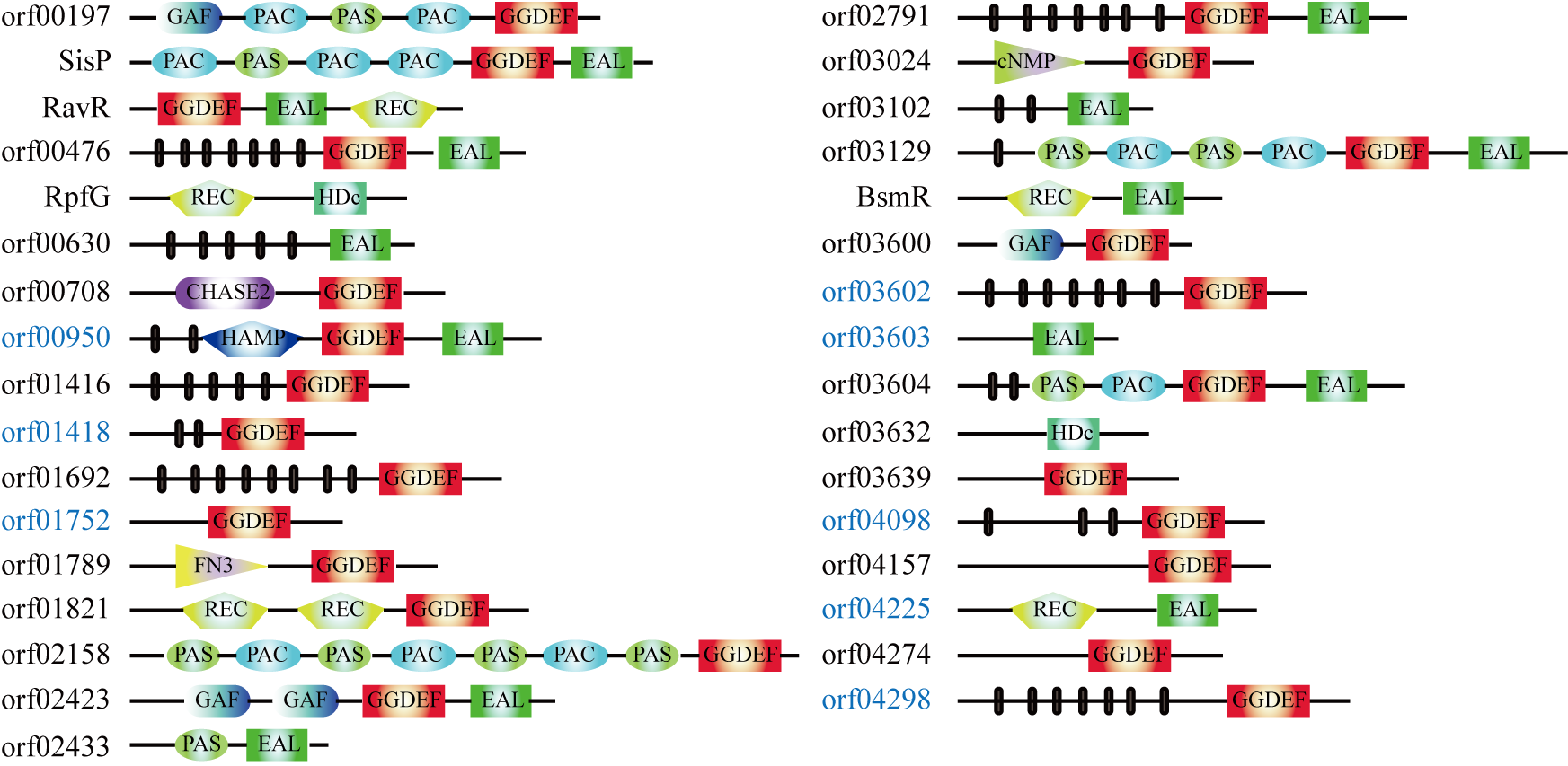

Supplement: FIG S1 [file mbio.01414-22-s0001.tif]

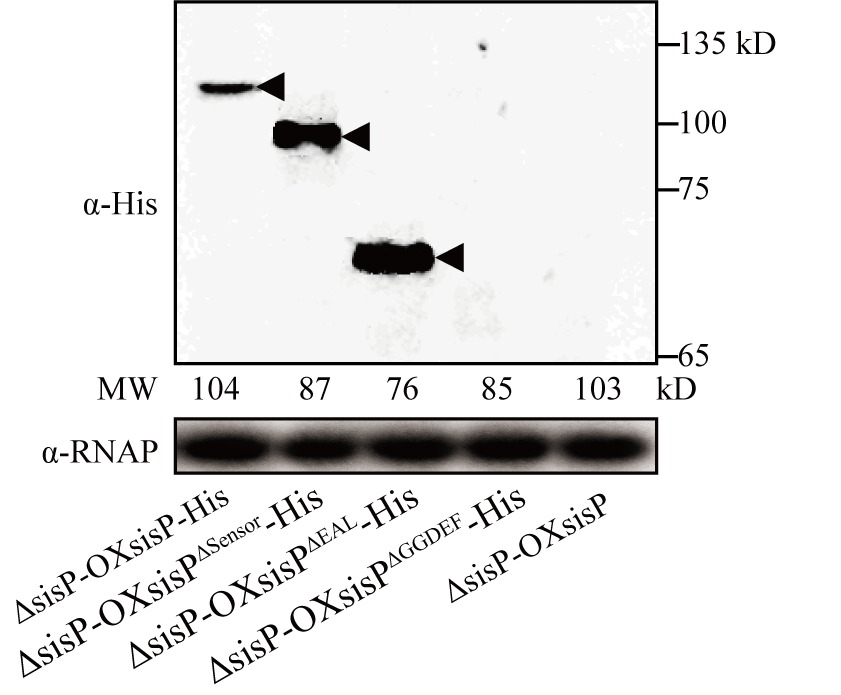

Supplement: FIG S2 [file mbio.01414-22-s0002.tif]

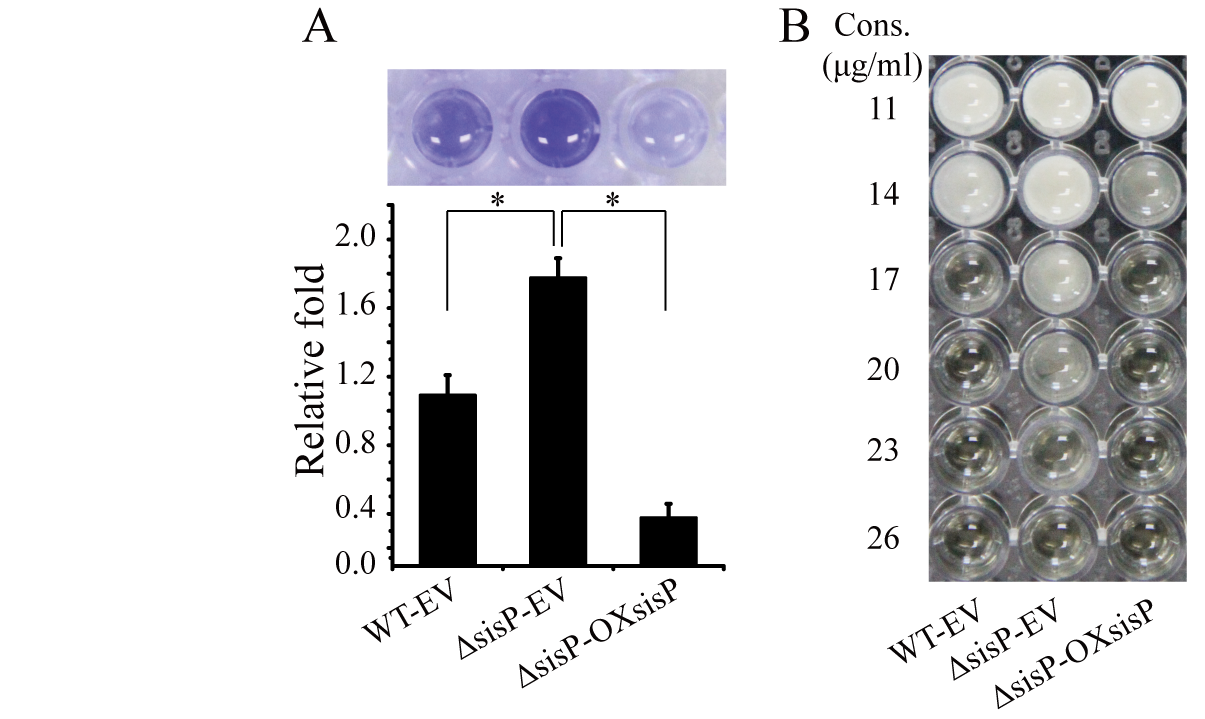

Supplement: FIG S3 [file mbio.01414-22-s0003.tif]

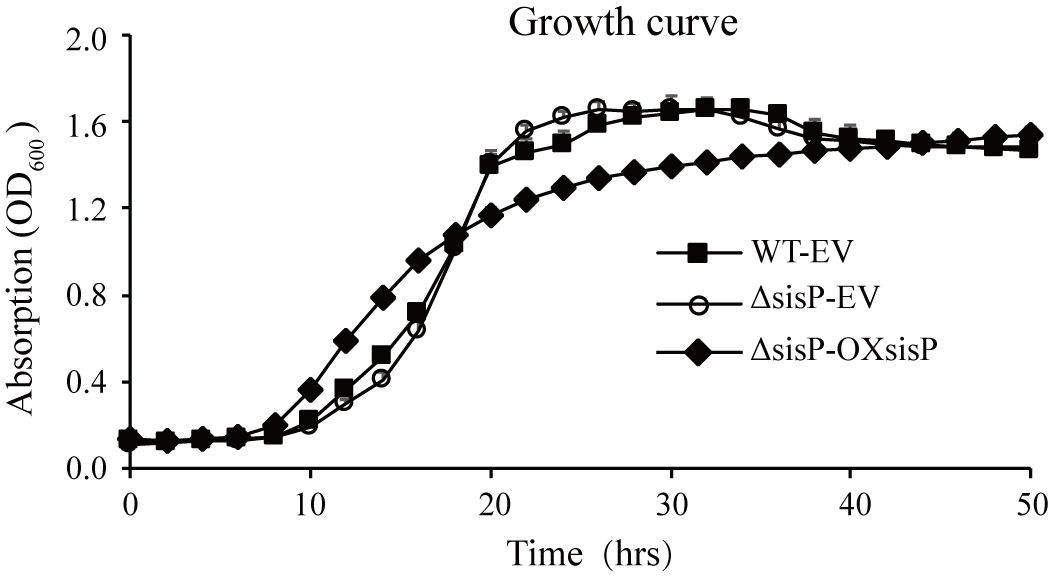

Supplement: FIG S4 [file mbio.01414-22-s0004.tif]

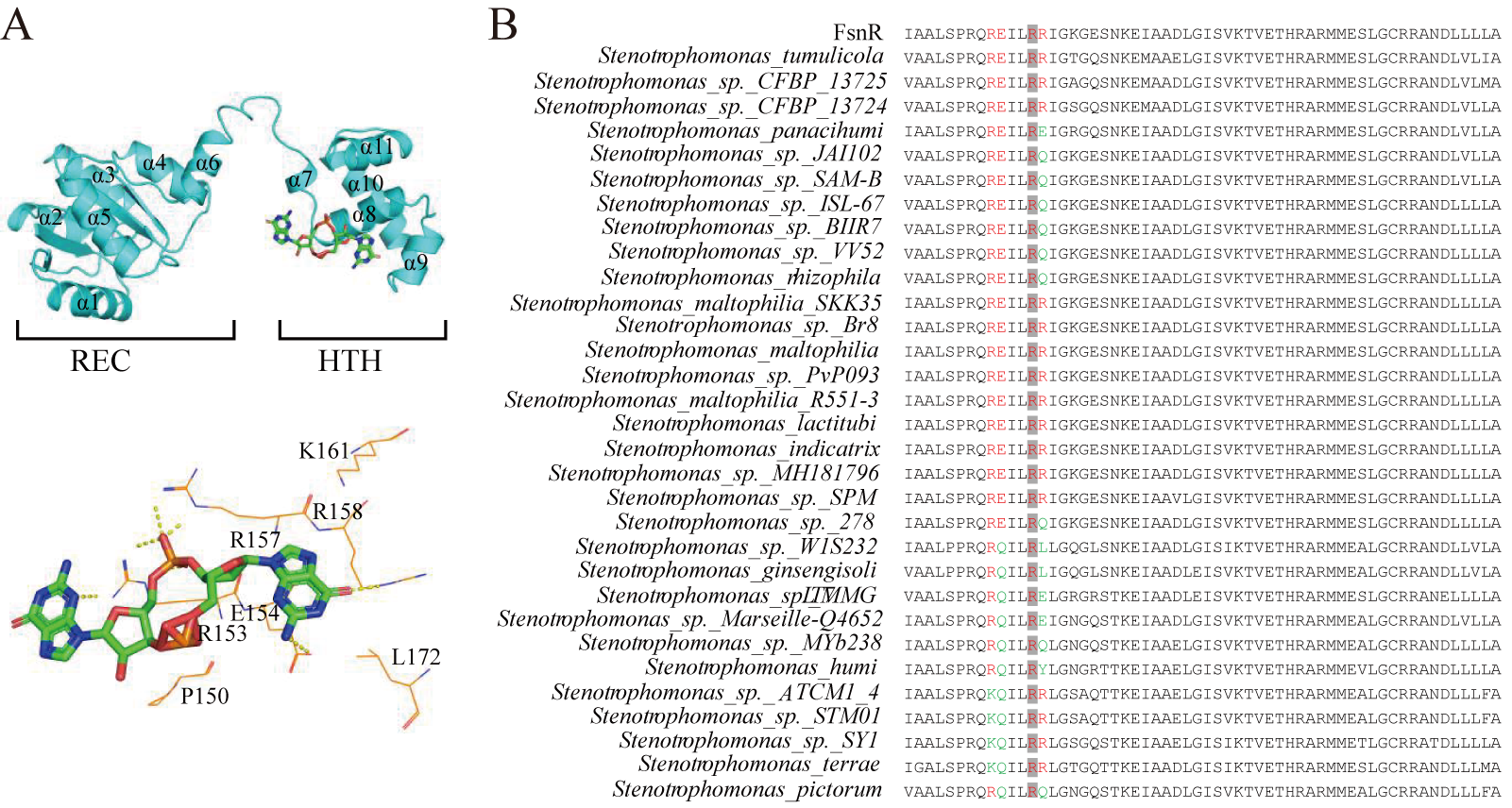

Supplement: FIG S5 [file mbio.01414-22-s0005.tif]

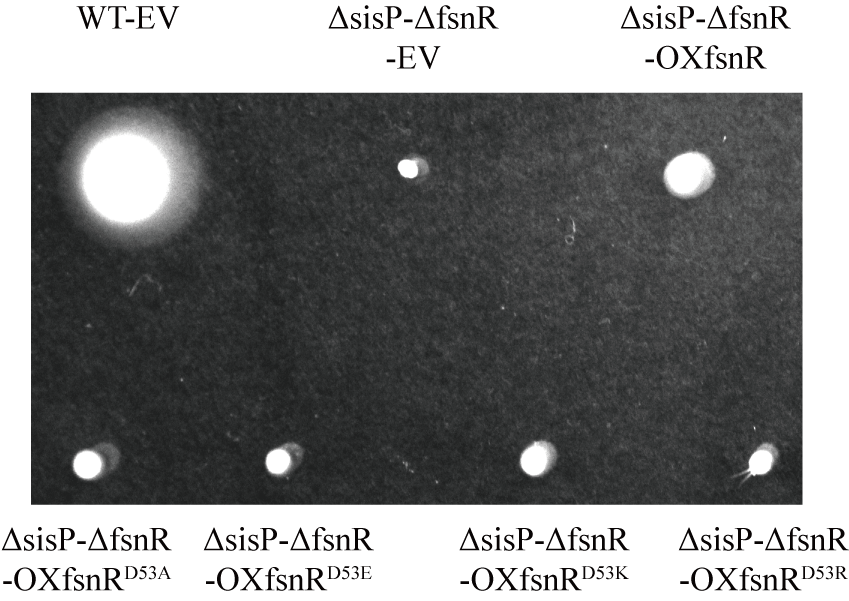

Supplement: FIG S6 [file mbio.01414-22-s0006.tif]

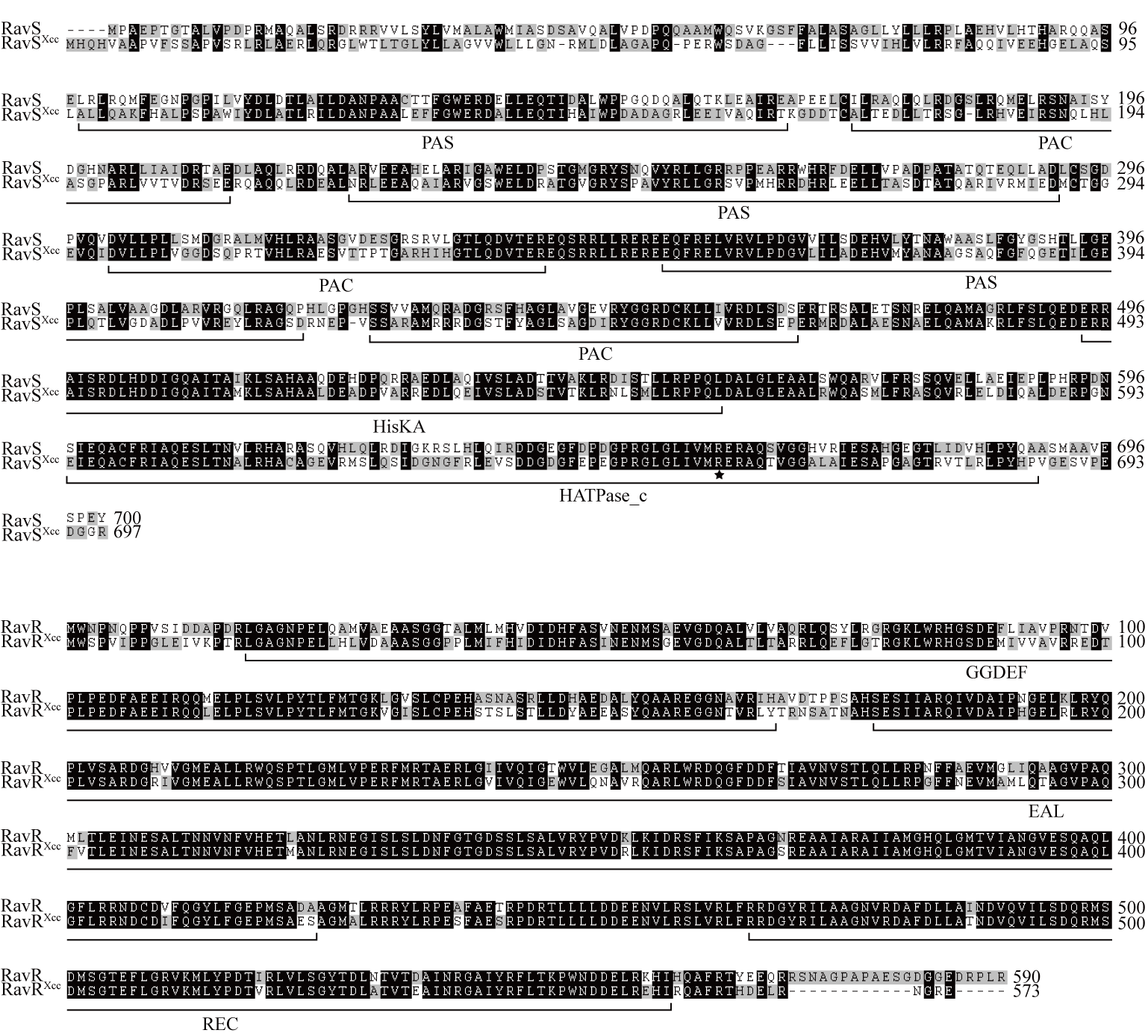

Supplement: FIG S7 [file mbio.01414-22-s0007.tif]
