# Supplementary material for: Dual Regulatory Role Exerted by Cyclic Dimeric GMP To Control FsnR-Mediated Bacterial Swimming
Source: mBio. 2022 Sep 7;13(5):e01414-22. doi: 10.1128/mbio.01414-22 (PMC9600515; doi:10.1128/mbio.01414-22)
Supplement: TABLE S1 [file mbio.01414-22-s0008.docx]

**Table S1. Bacterial strains and plasmids used in this study.**

| **Strain or plasmid** | **Genotype or description** | **Source** |
| --- | --- | --- |
| **Strains** |  |  |
| *E. coli* DH5α | Host strain used for molecular cloning | Lab collection |
| *E. coli* BL21(DE3) | Host strain used for protein expression | Lab collection |
| *S. maltophilia* CGMCC 1.1788 | Wild type strain (WT) | Lab collection |
| WT-pBBR1MCS2 | *S. maltophilia* wild-type strain containing blank pBBR1MCS2 vector | This study |
| Δ*sisP*-pBBR1MCS2 | In-frame deletion mutant of orf00430 containing blank pBBR1MCS2 vector | This study |
| Δ*sisP*-OX*sisP* | Complementary strain with *sisP* constitutively expressed in Δ*sisP* mutant | This study |
| Δ*sisP*-OX*sisP*^Δsensor^ | Complementary strain with *sisP*^Δsensor^, constitutively expressed in Δ*sisP* mutant | This study |
| Δ*sisP*-OX*sisP*-His | Complementary strain with *sisP*-His constitutively expressed in Δ*sisP* mutant | This study |
| Δ*sisP*-OX*sisP*^Δsensor^-His | Complementary strain with *sisP*^Δsensor^-His, constitutively expressed in Δ*sisP* mutant | This study |
| Δ*sisP*-OX*sisP*^ΔGGDEF^-His | Complementary strain with *sisP*^ΔGGDEF^-His, constitutively expressed in Δ*sisP* mutant | This study |
| Δ*sisP*-OX*sisP*^ΔEAL^-His | Complementary strain with *sisP*^ΔEAL^-His, constitutively expressed in Δ*sisP* mutant | This study |
| Δ*sisP*-OX*pcrK* | Complementary strain with *pcrK* constitutively expressed in Δ*sisP* mutant | This study |
| Δ*sisP*-OX*fsnR* | Complementary strain with *fsnR* constitutively expressed in Δ*sisP* mutant | This study |
| Δ*sisP*-OX*fsnR*^D53A^ | Complementary strain with *fsnR*^D53A^ constitutively expressed in Δ*sisP* mutant | This study |
| Δ*ravS*-pBBR1MCS2 | In-frame deletion mutant of orf00431 containing blank pBBR1MCS2 vector | This study |
| Δ*fsnR*-pBBR1MCS2 | In-frame deletion mutant of orf00457 containing blank pBBR1MCS2 vector | This study |
| Δ*fsnR*-OX*fsnR* | Complementary strain with *fsnR* constitutively expressed in Δ*fsnR* mutant | This study |
| Δ*fsnR*-OX*fsnR*^D53A^ | Complementary strain with *fsnR*^D53A^ constitutively expressed in Δ*fsnR* mutant | This study |
| Δ*fsnR*-OX*fsnR*^R157A^ | Complementary strain with *fsnR*^R157A^ constitutively expressed in Δ*fsnR* mutant | This study |
| Δ*sisP-*Δ*ravS*-pBBR1MCS2 | Double in-frame deletion mutant of orf00430 and orf00431 containing blank pBBR1MCS2 vector | This study |
| Δ*sisP-*Δ*ravS*-OX*sisP* | Epistasis analysis strain with *sisP* constitutively expressed in Δ*sisP-*Δ*ravS* mutant | This study |
| Δ*sisP-*Δ*ravS*-OX*ravS* | Epistasis analysis strain with *ravS* constitutively expressed in Δ*sisP-*Δ*ravS* mutant | This study |
| Δ*sisP-*Δ*ravS*-OX*ravS*^H503A^ | Epistasis analysis strain with *ravS*^H503A^ constitutively expressed in Δ*sisP-*Δ*ravS* mutant | This study |
| Δ*sisP-*Δ*ravS*-OX*fsnR* | Epistasis analysis strain with *fsnR* constitutively expressed in Δ*sisP-*Δ*ravS* mutant | This study |
| Δ*sisP-*Δ*ravS-*OX*fsnR*^D53A^ | Epistasis analysis strain with *fsnR*^D53A^ constitutively expressed in Δ*sisP-*Δ*ravS* mutant | This study |
| Δ*sisP-*Δ*fsnR*-pBBR1MCS2 | Double in-frame deletion mutant of orf00430 and orf00457 containing blank pBBR1MCS2 vector | This study |
| Δ*sisP-*Δ*fsnR*-OX*sisP* | Epistasis analysis strain with *sisP* constitutively expressed in Δ*sisP-*Δ*fsnR* mutant | This study |
| Δ*sisP-*Δ*fsnR*-OX*fsnR* | Epistasis analysis strain with *fsnR* constitutively expressed in Δ*sisP-*Δ*fsnR* mutant | This study |
| Δ*sisP-*Δ*fsnR*-OX*fsnR*^D53A^ | Epistasis analysis strain with *fsnR*^D53A^ constitutively expressed in Δ*sisP-*Δ*fsnR* mutant | This study |
| Δ*sisP-*Δ*fsnR*-OX*fsnR*^D53E^ | Epistasis analysis strain with *fsnR*^D53E^ constitutively expressed in Δ*sisP-*Δ*fsnR* mutant | This study |
| Δ*sisP-*Δ*fsnR*-OX*fsnR*^D53K^ | Epistasis analysis strain with *fsnR*^D53K^ constitutively expressed in Δ*sisP-*Δ*fsnR* mutant | This study |
| Δ*sisP-*Δ*fsnR*-OX*fsnR*^D53R^ | Epistasis analysis strain with *fsnR*^D53R^ constitutively expressed in Δ*sisP-*Δ*fsnR* mutant | This study |
| Δ*sisP-*Δ*fsnR*-OX*fsnR*^R157A^ | Epistasis analysis strain with *fsnR*^R157A^ constitutively expressed in Δ*sisP-*Δ*fsnR* mutant | This study |
| DEL00197 | Insertional inactivation mutant of *orf00197* | This study |
| DEL00430 | Insertional inactivation mutant of *sisP* | This study |
| DEL00432 | Insertional inactivation mutant of *ravR* | This study |
| DEL00476 | Insertional inactivation mutant of *orf00476* | This study |
| DEL00516 | Insertional inactivation mutant of *rpfG* | This study |
| DEL00630 | Insertional inactivation mutant of *orf00630* | This study |
| DEL00708 | Insertional inactivation mutant of *orf00708* | This study |
| DEL00950 | Insertional inactivation mutant of *orf00950* | This study |
| DEL01416 | Insertional inactivation mutant of *orf01416* | This study |
| DEL01418 | Insertional inactivation mutant of *orf01418* | This study |
| DEL01692 | Insertional inactivation mutant of *orf01692* | This study |
| DEL01752 | Insertional inactivation mutant of *orf01752* | This study |
| DEL01789 | Insertional inactivation mutant of *orf01789* | This study |
| DEL01821 | Insertional inactivation mutant of *orf01821* | This study |
| DEL02158 | Insertional inactivation mutant of *orf02158* | This study |
| DEL02423 | Insertional inactivation mutant of *orf02423* | This study |
| DEL02433 | Insertional inactivation mutant of *orf02433* | This study |
| DEL02791 | Insertional inactivation mutant of *orf02791* | This study |
| DEL03024 | Insertional inactivation mutant of *orf03024* | This study |
| DEL03102 | Insertional inactivation mutant of *orf03102* | This study |
| DEL03129 | Insertional inactivation mutant of *orf03129* | This study |
| DEL03262 | Insertional inactivation mutant of *orf03262* | This study |
| DEL03600 | Insertional inactivation mutant of *orf03600* | This study |
| DEL03602 | Insertional inactivation mutant of *orf03602* | This study |
| DEL03603 | Insertional inactivation mutant of *orf03603* | This study |
| DEL03604 | Insertional inactivation mutant of *orf03604* | This study |
| DEL03632 | Insertional inactivation mutant of *orf03632* | This study |
| DEL03639 | Insertional inactivation mutant of *orf03639* | This study |
| DEL04098 | Insertional inactivation mutant of *orf04098* | This study |
| DEL04157 | Insertional inactivation mutant of *orf04157* | This study |
| DEL04225 | Insertional inactivation mutant of *orf04225* | This study |
| DEL04274 | Insertional inactivation mutant of *orf04274* | This study |
| DEL04298 | Insertional inactivation mutant of *orf04298* | This study |
| **Plasmids** |  |  |
| pK18mob | Suicide vector used in single-crossover recombination, Kan^r^ | This study |
| pK18mobsacB | Suicide vector used in double-crossover recombination, Kan^r^ | This study |
| pBBR1MCS2 | Broad-host-range vector used for genetic complementation, Kan^r^ | This study |
| pET30a | Protein expression vector, Kan^r^ | This study |
| pGEX-6P-1 | Protein expression vector, Amp^r^ | This study |
| pK18mob::00197 | For insertional inactivation of *orf00197* | This study |
| pK18mob::00430 | For insertional inactivation of *sisP* | This study |
| pK18mob::00432 | For insertional inactivation of *ravR* | This study |
| pK18mob::00476 | For insertional inactivation of *orf00476* | This study |
| pK18mob::00516 | For insertional inactivation of *rpfG* | This study |
| pK18mob::00630 | For insertional inactivation of *orf00630* | This study |
| pK18mob::00708 | For insertional inactivation of *orf00708* | This study |
| pK18mob::00950 | For insertional inactivation of *orf00950* | This study |
| pK18mob::01416 | For insertional inactivation of *orf01416* | This study |
| pK18mob::01418 | For insertional inactivation of *orf01418* | This study |
| pK18mob::01692 | For insertional inactivation of *orf01692* | This study |
| pK18mob::01752 | For insertional inactivation of *orf01752* | This study |
| pK18mob::01789 | For insertional inactivation of *orf01789* | This study |
| pK18mob::01821 | For insertional inactivation of *orf01821* | This study |
| pK18mob::02158 | For insertional inactivation of *orf02158* | This study |
| pK18mob::02423 | For insertional inactivation of *orf02423* | This study |
| pK18mob::02433 | For insertional inactivation of *orf02433* | This study |
| pK18mob::02791 | For insertional inactivation of *orf02791* | This study |
| pK18mob::03024 | For insertional inactivation of *orf03024* | This study |
| pK18mob::03102 | For insertional inactivation of *orf03102* | This study |
| pK18mob::03129 | For insertional inactivation of *orf03129* | This study |
| pK18mob::03262 | For insertional inactivation of *orf03262* | This study |
| pK18mob::03600 | For insertional inactivation of *orf03600* | This study |
| pK18mob::03602 | For insertional inactivation of *orf03602* | This study |
| pK18mob::03603 | For insertional inactivation of *orf03603* | This study |
| pK18mob::03604 | For insertional inactivation of *orf03604* | This study |
| pK18mob::03632 | For insertional inactivation of *orf03632* | This study |
| pK18mob::03639 | For insertional inactivation of *orf03639* | This study |
| pK18mob::04098 | For insertional inactivation of *orf04098* | This study |
| pK18mob::04157 | For insertional inactivation of *orf04157* | This study |
| pK18mob::04225 | For insertional inactivation of *orf04225* | This study |
| pK18mob::04274 | For insertional inactivation of *orf04274* | This study |
| pK18mob::04298 | For insertional inactivation of *orf04298* | This study |
| pK18mobsacB::00430 | For *sisP* in-frame deletion construction | This study |
| pK18mobsacB::00431 | For *ravS* in-frame deletion construction | This study |
| pK18mobsacB::00457 | For *fsnR* in-frame deletion construction | (Kang *et al*, 2015) |
| pET30a-SisP | Protein expression vector, pET30a::SisP,expressing full-length SisP | This study |
| pET30a-RavS | Protein expression vector, pET30a::RavS,expressing full-length RavS | This study |
| pET30a-RavS^H503A^ | Protein expression vector, pET30a::RavS^H503A^,expressing full-length RavS^H503A^ | This study |
| pET30a-RavR | Protein expression vector,pET30a::RavR,expressing full-length RavR | This study |
| pET30a-RavR^D496A^ | Protein expression vector, pET30a::RavR^D496A^,expressing full-length RavR^D496A^ | This study |
| pET30a-FsnR | Protein expression vector, pET30a::FsnR,expressing full-length FsnR | (Kang *et al*, 2015) |
| pGEX-6P-1-SisP^ΔEAL^ | Protein expression vector, pGEX-6P-1::SisP^ΔEAL^, expressing SisP without sensor domain | This study |
| pGEX-6P-1-SisP^Δsensor^ | Protein expression vector, pGEX-6P-1::SisP^Δsensor^, expressing SisP without sensor domain | This study |
| pGEX-6P-1-SisP-sensor | Protein expression vector, pGEX-6P-1::SisP-sensor, expressing the sensor domain of SisP | This study |
| pGEX-6P-1-FsnR-LuxR | Protein expression vector, pGEX-6P-1::FsnR-LuxR, expressing the LuxR domain protein of FsnR | This study |
| pGEX-6P-1-FsnR-REC | Protein expression vector, pGEX-6P-1::REC, expressing the REC domain protein of FsnR | This study |
| pGEX-6P-1-FsnR-LuxR^R157A^ | Protein expression vector, pGEX-6P-1::FsnR-LuxR^R157A^, expressing the LuxR domain protein of FsnR with Arg^157^ mutated into Ala | This study |
| pGEX-6P-1-DncV | Protein expression vector, pGEX-6P-1:: DncV, expressing full-length DncV | This study |
| pBBR1MCS2::*sisP* | Genetic complementation vector, pBBR1MCS2::sisP | This study |
| pBBR1MCS2::*sisP*^Δsensor^ | Genetic complementation vector, pBBR1MCS2::*sisP*^Δsensor^ | This study |
| pBBR1MCS2::*sisP*-His | Genetic complementation vector, pBBR1MCS2::sisP-His | This study |
| pBBR1MCS2::*sisP*^Δsensor^-His | Genetic complementation vector, pBBR1MCS2::*sisP*^Δsensor^-His | This study |
| pBBR1MCS2::*sisP*^ΔGGDEF^-His | Genetic complementation vector, pBBR1MCS2::*sisP*^ΔGGDEF^-His | This study |
| pBBR1MCS2::*sisP*^ΔEAL^-His | Genetic complementation vector, pBBR1MCS2::*sisP*^ΔEAL^-His | This study |
| pBBR1MCS2::*pcrK* | Genetic complementation vector, pBBR1MCS2::*pcrK* | This study |
| pBBR1MCS2::*ravS* | Genetic complementation vector, pBBR1MCS2::*ravS* | This study |
| pBBR1MCS2::*ravS*^H503A^ | Genetic complementation vector, pBBR1MCS2::*ravS*^H503A^ | This study |
| pBBR1MCS2::*fsnR* | Genetic complementation vector, pBBR1MCS2::*fsnR* | This study |
| pBBR1MCS2::*fsnR*^D53A^ | Genetic complementation vector, pBBR1MCS2::*fsnR*^D53A^ | This study |
| pBBR1MCS2::*fsnR*^D53E^ | Genetic complementation vector, pBBR1MCS2::*fsnR*^D53E^ | This study |
| pBBR1MCS2::*fsnR*^D53K^ | Genetic complementation vector, pBBR1MCS2::*fsnR*^D53K^ | This study |
| pBBR1MCS2::*fsnR*^D53R^ | Genetic complementation vector, pBBR1MCS2::*fsnR*^D53R^ | This study |
| pBBR1MCS2::*fsnR*^R157A^ | Genetic complementation vector, pBBR1MCS2::*fsnR*^R157A^ | This study |

Kan^r^ : kanamycin resistant; Amp^r^: Ampicillin resistant; CGMCC: China General Microbiological Culture Collection Center.
