# Supplementary material for: Dual Regulatory Role Exerted by Cyclic Dimeric GMP To Control FsnR-Mediated Bacterial Swimming
Source: mBio. 2022 Sep 7;13(5):e01414-22. doi: 10.1128/mbio.01414-22 (PMC9600515; doi:10.1128/mbio.01414-22)
Supplement: TABLE S2 [file mbio.01414-22-s0009.docx]

**Table S2. Primers used in this study.**

| **Primer** | **Sequence (5′-3′)** | **Description** |
| --- | --- | --- |
| ΔsisP | W: CCCAAGCTTGTGGACCAAGGGATCATC  X: CCGCACGATCTCGCCGCACAGCCCTTCCAGCTGCTT  Y: AAGCAGCTGGAAGGGCTGTGCGGCGAGATCGTGCGG  Z: CCGGAATTCTCACCGCATCAGCATCAC | For Δ*sisP* construction |
| OXsisP | W: CCCAAGCTTGTGGACCAAGGGATCATC  Z: CCGGAATTCTCACCGCATCAGCATCAC | For *sisP* complementation |
| OX-His-sisP | W: CCCAAGCTTGTGGACCAAGGGATCATC  Z: GGACTAGTTCAATGATGGTGATGATGGTGCCGCATCAGCA  TCACTTC | For *sisP*-His complementation |
| OX-His-sisPΔsensor | W: CCCAAGCTTGTGGACCAAGGGATCATC  X: CTCCTCGGTCCACTGCATCTGCTGTTCCTGCAGCAG  Y: CTGCTGCAGGAACAGCAGATGCAGTGGACCGAGGAG  Z: GGACTAGTTCAATGATGGTGATGATGGTGCCGCATCAGCA  TCACTTC | For *sisP*^Δsensor^-His complementation |
| OX-His-sisPΔGGDEF | W: CCCAAGCTTGTGGACCAAGGGATCATC  X: GGCCAATGCGTCCTGGCTGCGGGTGATGTCACGGAA  Y: TTCCGTGACATCACCCGCAGCCAGGACGCATTGGCC  Z: GGACTAGTTCAATGATGGTGATGATGGTGCCGCATCAGCA  TCACTTC | For *sisP*^ΔGGDEF^-His complementation |
| OX-His-sisPΔEAL | W: CCCAAGCTTGTGGACCAAGGGATCATC  X: GCGCAGCCACTGCGCCAGCTGGATGCGGGCCAATGC  Y: GCATTGGCCCGCATCCAGCTGGCGCAGTGGCTGCGC  Z: GGACTAGTTCAATGATGGTGATGATGGTGCCGCATCAGCA  TCACTTC | For *sisP*^ΔEAL^-His complementation |
| Pro-His-SisP | F: CGCCATATGGACCAAGGGATCATCGCCA  R: CCCAAGCTTCCGCATCAGCATCACTTCC | For SisP expression |
| Pro-GST-SisP-sensor | F: CGCGGATCCGACATCGCCCAGGTGCGGG  R: TTGCGGCCGCGGGCAGGCCGGTCAGCAGA | For SisP-sensor expression |
| Pro-GST-SisPΔsensor | F: CGCGGATCCGACCAAGGGATCATCGCCA  X: CTCCTCGGTCCACTGCATCTGCTGTTCCTGCAGCAG  Y: CTGCTGCAGGAACAGCAGATGCAGTGGACCGAGGAG  R: TTGCGGCCGCCCGCATCAGCATCACTTCC | For SisP-Δsensor expression |
| ΔravS | W: GAATTCCAGCGTAAAGGCCAGCAGG  X: CCATGGAGCAGGCGGCGATGTGG  Y: CCATGGGTGCAACTGCGCGACATCG  Z: AAGCTTGTCGGTGTTGCGCGGGA | For Δ*ravS* construction |
| OXravS | W: CCCAAGCTTATGCCTGCAGAACCGACC  Z: CCGGAATTCTCAGTATTCCGGTGACTC | For *ravS* complementation |
| RavS-H503A | F: GCGATCTGGCCGACGACATCGGCCAGG  R: GTCGTCGGCCAGATCGCGCGAGATCGC | For *ravS*^H503A^ construction and RavS^H503A^ expression |
| Pro-His-RavS | F: CGCCATATGCCTGCAGAACCGACCGGC  R: CCCAAGCTTGTATTCCGGTGACTCAAC | For RavS expression |
| Pro-His-RavR | F: CGCCATATGTGGAATCCCAACCAGCCGC  R: ATTTGCGGCCGCCCGCAGCGGGCGATCCTC | For RavR expression |
| RavR-D496A | F: CGTGCAGGTGATCCTGTCCGCCCAGCGCATGAGCGACAT  G  R: CATGTCGCTCATGCGCTGGGCGGACAGGATCACCTGCAC  G | For RavR^D496A^ expression |
| FsnR-D53A | W: CCGGAATTCGTGCGAGTTCTCATCGTC  Z: CGCGGATCCTTACTCCAGCTCGTGCTG | For *fsnR*^D53A^ construction |
| FsnR-  D53E | W: CGTGGTCCTGATGGAACTGTCATTGCCTGGC  Z: GCCAGGCAATGACAGTTCCATCAGGACCACG | For *fsnR*^D53E^ construction |
| FsnR-D53K | W: CGTGGTCCTGATGAAGCTGTCATTGCCTGGC  Z: GCCAGGCAATGACAGCTTCATCAGGACCACG | For *fsnR*^D53K^ construction |
| FsnR-D53R | W: CGTGGTCCTGATGCGCCTGTCATTGCCTGGC  Z: GCCAGGCAATGACAGGCGCATCAGGACCACG | For *fsnR*^D53R^ construction |
| FsnR-R157A | F: CGTGGTCCTGATGGCCCTGTCATTGCCTGGC  R: GGCCATCAGGACCACGTCCGGGGCGTGCTGC | For *fsnR*^R157A^ construction |
| Pro-His-FsnR | F: CAGCGCGAGATCCTGGCCCGCATCGGCA  R: GGCCAGGATCTCGCGCTGGCGCGGCGAC | For FsnR expression |
| Pro-GST-FsnR-REC | F: CGCCATATGCGAGTTCTCATCGTCGACG  R: CCCAAGCTTCTCCAGCTCGTGCTGATGG | For FsnR-REC expression |
| Pro-GST-FsnR-LuxR | F: CGCGGATCCCGAGTTCTCATCGTCGAC  R: CCGGAATTCTTACAGGAACACCTGGCCGGC | For FsnR-LuxR expression |
| Pro-GST-FsnR-LuxR^R157A^ | F: CGTGGTCCTGATGGCCCTGTCATTGCCTGGC  R: GGCCATCAGGACCACGTCCGGGGCGTGCTGC | For FsnR- LuxR^R157A^ expression |
| Pro-GST-DncV | F: CGCGGATCCCCGATGCTGGGCCGCGAG  R: CCGGAATTCTTACTCCAGCTCGTGCTGATG | For DncV expression |
| OXpcrK | W: CCAAGCTTATGCCAGCCCGTCCCCTG  Z: CGGAATTCTCAACGCTCCCGATCCCAG | For *pcrK* complementation |
| P0453 | F: CGCGGATCCAGAATGACTTGGAACTTT  R: ACGCGTCGACTCAGCCACTTACCATTGTGCT | For PCR of *fliD* promoter region |
| 00427 | F: CAACGAGATCGAGGGTTC  R: GTGCTGAGCCATCTGGTC | For qRT-PCR of *mcp* |
| 00436 | F: ATGAGCCAGAAAATCGAC  R: GTGCAGCTGCTGGTCCAG | For qRT-PCR of *flgM* |
| 00439 | F: GTTGCGCGAACAGCGGAT  R: ACCTTGCTCTCGACGAAG | For qRT-PCR of *flgB* |
| 00450 | F: CTGCAGGCCGAAGTGACC  R: AACCTTGATCGTGGTGGC | For qRT-PCR of *fliC* |
| 00453 | F: CAAGATGCAGCTGTCGTC  R: GAACGTGTCGGTCTTCGG | For qRT-PCR and ChIP-qPCR of *fliD* |
| 00460 | F: CTGGAAAGCGAACTGTTC  R: GGTAGAACAGATCCTCGC | For qRT-PCR of *flrA* |
| 00468 | F: GTCGACCACTGACAGCAG  R: CTGGTCGCCGCCGGTTTG | For qRT-PCR of *fliK* |
| 00469 | F: TCCAAGCACGAAGACAAG  R: GGTCTCGGCCTTTAGCAC | For qRT-PCR of *fliL* |
| 00481 | F: TGGGCAAGACGAATGTGT  R: TTCGGGCCTTCGACGATG | For qRT-PCR of *flhG* |
| 00486 | F: GTCGAGTGGAGCAACATC  R: GAGTTTGCTCGGGTCGGC | For qRT-PCR of *motA* |
| tmRNA | F: GGGGGTGCACTGGTTTCG  R: TGGTGGAGGTGGGCGGAAT | For qRT-PCR of *tmRNA* |
| 00197 | F: CGGAATTCGGACGAAGCCGAACGACTG  R: TTGCGGCCGCTCAACGCGCGGCAGGGTC | For pK18mob::00197 construction |
| 00430 | F: CGGGATCCGGACCAAGGGATCATCGCC  R: TTGCGGCCGCTCACCGCATCAGCATCAC | For pK18mob::sisP construction |
| 00432 | F: CGGGATCCGTGGAATCCCAACCAGCCG  R: TTGCGGCCGCTTACCGCAGCGGGCGATC | For pK18mob::ravR construction |
| 00476 | F: CGGGATCCGCTGGTAGGCACGTACAAC  R: TTGCGGCCGCCTACAGGGGGGCGTTGTC | For pK18mob::00476 construction |
| 00516 | F: CCGGAATTCACCCGGTCGACCTGCTGC  R: CCCAAGCTTGGCTGCCACTGAGCAGCT | For pK18mob::00516 construction |
| 00630 | F: CCGGAATTCATCGATCCGATCAGTGGC  R: CCCAAGCTTTGCTTGGGCTGGTAGCAC | For pK18mob::00630 construction |
| 00708 | F: CGAGCTCGGAGGCGCTCGCCGCTGCCG  R: TTGCGGCCGCTCACGCGCCGCCGTCGAT | For pK18mob::00708 construction |
| 00950 | F: CGGAATTCGAAGTGGCTCGGCTCGGGG  R: TTGCGGCCGCTCAAACGCCCGATTCGAT | For pK18mob::00950 construction |
| 01416 | F: CGGGATCCGCCATTCTTCACCGATTCG  R: TTGCGGCCGCTCACTCGCCGTAGCTGGC | For pK18mob::01416 construction |
| 01418 | F: CGAGCTCGGTGCATGGCCCGCAAGGCG  R: TTGCGGCCGCTCAACGCGTGATGCGACC | For pK18mob::01418 construction |
| 01692 | F: CGGGATCCGCTGGCGCTGCTGTATGTG  R: TTGCGGCCGCTCAGCAGTCCATCGTTCC | For pK18mob::01692 construction |
| 01752 | F: CGGGATCCGCCTCCCGAGCTGACAGCT  R: TTGCGGCCGCTCAGCCCAGCTCGGCGCC | For pK18mob::01752 construction |
| 01789 | F: CGGAATTCGGAGCGCCGCGTTTCGAAG  R: TTGCGGCCGCTCAACCAATACTCGTACG | For pK18mob::01789 construction |
| 01821 | F: CGAGCTCGGAGCCACCGCATCATGCCC  R: TTGCGGCCGCTCAGGGCAGCGCCGGCTC | For pK18mob::01821 construction |
| 02158 | F: CGGAATTCGGCGGGCCAGGGCACCAAC  R: TTGCGGCCGCTCAGCCCTCGACGACCTC | For pK18mob::02158 construction |
| 02423 | F: CGGGATCCGGAGCCCGCCACCGGCGAT  R: TTGCGGCCGCTCAGCGTGCTGATCGGGA | For pK18mob::02423 construction |
| 02433 | F: CGGGATCCGCAGAACGCGAACGACATC  R: TTGCGGCCGCTCACCCGAACTCGAACCC | For pK18mob::02433 construction |
| 02791 | F: CGGGATCCGACTGGCAGTTACAGTCAG  R: TTGCGGCCGCTCACCCCACCCGGCGCAG | For pK18mob::02791 construction |
| 03024 | F: CGGGATCCGACCGGCAGGGGTGCGGCC  R: TTGCGGCCGCCTAGGGTGAGTCCACTGC | For pK18mob::03024 construction |
| 03102 | F: CCGGAATTCGGACGGCCGCCTCCTGGC  R: CCCAAGCTTATCATCTTCAGCGCACGG | For pK18mob::03102 construction |
| 03129 | F: CGGGATCCGGCGATCGTGCTGGCCTAC  R: TTGCGGCCGCTTAGGCGGCCACCTCATC | For pK18mob::03129 construction |
| 03262 | F: CCGGAATTCCGCTGACGTGGACGCGCT  R: CCCAAGCTTGGCATAGAAGTCGCTGAC | For pK18mob::03262 construction |
| 03600 | F: CGGAATTCGATCAAGCCCGACAAGCCT  R: TTGCGGCCGCTCAGCCCGCCAGCTCCAC | For pK18mob::03600 construction |
| 03602 | F: CGGGATCCGGTGTGGTTGGCGTTGCCG  R: TTGCGGCCGCTCATGCCAGGGCGGGCGG | For pK18mob::03602 construction |
| 03603 | F: CCGGAATTCCCAGTTGCCGGCGGACAT  R: CCCAAGCTTGATCGACCAGGGTCAGCC | For pK18mob::03603 construction |
| 03604 | F: CGGGATCCGCCGATGACCCGTGGCCTG  R: TTGCGGCCGCTCACCGCATCGATGAAGG | For pK18mob::03604 construction |
| 03632 | F: CCGGAATTCTCTCGATGTCGCTGCAGA  R: CCCAAGCTTTTCCATCGCCGCGCGGTA | For pK18mob::03632 construction |
| 03639 | F: CGGGATCCGGACGATGCCAGCCAGCCG  R: TTGCGGCCGCTCATCGCTGCAGCGCACG | For pK18mob::03639 construction |
| 04098 | F: CGGGATCCGCGAACGCTGTCTCCCCGC  R: TTGCGGCCGCTCAATCGGCGTAGGGGTA | For pK18mob::04098 construction |
| 04157 | F: CGGGATCCGCAGCACGGTTTGCAGGGC  R: TTGCGGCCGCTTACACGGGCACGGCGTC | For pK18mob::04157 construction |
| 04225 | F: CCGGAATTCACCAGCAATCCGGAAACC  R: CCCAAGCTTGCGCGGTGTTGCCGCCGG | For pK18mob::04225 construction |
| 04274 | F: CGGGATCCGACGGACCAGCCCGACCAC  R: TTGCGGCCGCTCAGCCCGCCTGCACCCG | For pK18mob::04274 construction |
| 04298 | F: CGGGATCCGCCCCTGCATCCGCTCCGC  R: TTGCGGCCGCTCAGCCGCCGCGTGCCGC | For pK18mob::04298 construction |
